# Methane-derived microbial biostimulant reduces greenhouse gas emissions and improves rice yield

**DOI:** 10.3389/fpls.2024.1432460

**Published:** 2024-09-05

**Authors:** Sarma Rajeev Kumar, Einstein Mariya David, Gangigere Jagadish Pavithra, Gopalakrishnan Sajith Kumar, Kuppan Lesharadevi, Selvaraj Akshaya, Chavadi Basavaraddi, Gopal Navyashree, Panakanahalli Shivaramu Arpitha, Padmanabhan Sreedevi, Khan Zainuddin, Saiyyeda Firdous, Bondalakunta Ravindra Babu, Muralidhar Udagatti Prashanth, Ganesan Ravikumar, Palabhanvi Basavaraj, Sandeep Kumar Chavana, Vinod Munisanjeeviah Lakshmi Devi Kumar, Theivasigamani Parthasarathi, Ezhilkani Subbian

**Affiliations:** ^1^ String Bio Private Limited, Bangalore, India; ^2^ String Bio Private Limited, Centre for Cellular and Molecular Platforms, Bangalore, India; ^3^ VIT School of Agricultural Innovations and Advanced Learning (VAIAL), Vellore Institute of Technology, Vellore, India; ^4^ School of Biosciences and Technology (SBST), Vellore Institute of Technology, Vellore, India

**Keywords:** climate change, global warming potential, grain yield, methane, microbial biostimulant, nitrous oxide, rice

## Abstract

**Introduction:**

More than half of the world’s population consumes rice as their primary food. The majority of rice production is concentrated in Asia, with the top 10 rice-growing countries accounting for 84% of the world’s total rice cultivation. However, rice production is also strongly linked to environmental changes. Among all the global sources of greenhouse gas (GHG) emissions, paddy cultivation stands out as a significant contributor to global methane (CH_4_) and nitrous oxide (N_2_O) emissions. This contribution is expected to increase further with the projected increase of 28% in global rice output by 2050. Hence, modifications to rice management practices are necessary both to increase yield and mitigate GHG emissions.

**Methods:**

We investigated the effect of seedling treatment, soil application, and foliar application of a methane-derived microbial biostimulant on grain yield and GHG emissions from rice fields over three seasons under 100% fertilizer conditions. Further, microbial biostimulant was also tested under 75% nitrogen (N) levels to demonstrate its effect on grain yield. To understand the mechanism of action of microbial biostimulant on crop physiology and yield, a series of physiological, transcript, and metabolite analyses were also performed.

**Results:**

Our three-season open-field studies demonstrated a significant enhancement of grain yield, up to 39%, with a simultaneous reduction in CH_4_ (31%–60%) and N_2_O (34%–50%) emissions with the use of methane-derived microbial biostimulant. Under 75% N levels, a 34% increase in grain yield was observed with microbial biostimulant application. Based on the physiological, transcript, and metabolite analyses data, we were further able to outline the potential mechanisms for the diverse synergistic effects of methane-derived microbial biostimulant on paddy, including indole-3-acetic acid production, modulation of photosynthesis, tillering, and panicle development, ultimately translating to superior yield.

**Conclusion:**

The reduction in GHG emission and enhanced yield observed under both recommended and reduced N conditions demonstrated that the methane-derived biostimulant can play a unique and necessary role in the paddy ecosystem. The consistent improvements seen across different field trials established that the methane-derived microbial biostimulant could be a scalable solution to intensify rice productivity with a lower GHG footprint, thus creating a win–win–win solution for farmers, customers, and the environment.

## Introduction

By 2050, the global population is projected to reach 10 billion, which will require a 70% increase in food production (van Dijk et al., 2021). For instance, by 2050, the annual demand for cereals such as maize, rice, and wheat is projected to reach 3.3 billion tons, or 800 million tons more than 2014’s combined harvest (FAO, 2016). To ensure a food-secure future, global crop production must increase significantly, be climate-resilient, and reduce its environmental impact. The use of innovative technologies or approaches for achieving sustainable agriculture has been a topic of debate in the recent past.

Rice is one of the world’s top three staple crops and is closely connected with food security, economic growth, employment, culture, and regional peace. This crop is the frontrunner in the fight against global poverty and hunger and is essential for agricultural growth. However, rice paddies are also one of the most significant sources of methane (CH_4_) and nitrous oxide (N_2_O) emissions (Linquist et al., 2012; Carlson et al., 2017; Timilsina et al., 2020; Qian et al., 2023). Global average annual CH_4_ emissions from rice fields are 283 kg/ha (Qian et al., 2023), accounting for up to ~11% (~30 million metric tons) of total global CH_4_ emissions (Olivier and Peters, 2020), while average N_2_O emissions from rice fields are 1.7 kg/ha, accounting for 11% of global agricultural emissions (Islam et al., 2018; Win et al., 2021; Qian et al., 2023). CH_4_ sets the pace for warming in the near term, as it traps very large quantities of heat over a shorter period. Hence, curbing CH_4_ emissions is one of the fastest and most effective strategies to reduce the rate of warming and limit temperature rise to 1.5°C. Similarly, N_2_O is the third strongest contributor to radiative forcing from anthropogenic emissions and also a primary cause of stratospheric ozone depletion (Yang et al., 2020). Greenhouse gas (GHG) emissions from the agricultural sector in developing countries have attracted significant attention in international negotiations within the United Nations Framework Convention on Climate Change (UNFCCC) (Beddington et al., 2012). The agricultural sector has a unique potential to provide beneficial contributions to the global carbon budget through the use of new technologies and the adoption of alternative cultivation practices. Several international organizations advocate strategies to reduce CH_4_ and N_2_O emissions from rice cultivation. Alternative agronomic practices, such as alternative wetting and drying (AWD), direct seeded rice (DSR), and improved rice cultivars, have all been evaluated for their effectiveness in reducing GHG emissions (Yusuf et al., 2012; Bhatia et al., 2013; Xu et al., 2017; Oo et al., 2018; Liu et al., 2022; FAO, 2023). To date, while cultivation practices like AWD and DSR have demonstrated the potential to bring a reduction in CH_4_ emissions, the impact on N_2_O emission and yield is often contradictory (Carrijo et al., 2017; Xu et al., 2019). Also, some of these methodologies require specific infrastructures and considerable habit change for farmers, which has been a limiting factor for adoption. Hence, innovative, scalable, and sustainable solutions that can enhance grain yield while reducing the GHG emissions from rice are the need of the hour.

Agricultural inputs such as biostimulants have demonstrated the ability to increase yield, enhance resistance to abiotic stress, and improve competitiveness and sustainability. Biostimulants have a revolutionary impact, and unlike chemical fertilizers, they function in synergy to sustain crop resilience. Among the different classes of biostimulants, microbial-based biostimulants have emerged as highly valuable and robust agricultural inputs for improving plant yield (Castiglione et al., 2021; Hijri, 2023; Kaushal et al., 2023). Rice fields form an important niche for aerobic methanotrophs, where they oxidize a significant amount of the CH_4_ generated and thus play a key role in mitigating CH_4_ emission (Conrad and Rothfuss, 1991; Conrad, 2009; Zheng et al., 2024). A few studies have reported the plant growth-promoting properties of methanotrophs (Rani et al., 2021a; Mohite et al., 2023) as well as their ability to reduce GHG emission and improve yield in rice (Jeya Bharathi et al., 2018; Taopan et al., 2018; Davamani et al., 2020; Rani et al., 2021b). However, most of these studies were performed for one season or were confined to a small testing area. Moreover, none of these studies investigated molecular changes in the plant during methanotroph application. Here, we report data from a three-season open-field study on rice with a methane-derived microbial biostimulant. Two objectives were addressed regarding the effect of methane-derived microbial biostimulant in paddy: i) to assess the effect on grain yield and GHG emissions across three seasons and ii) to understand the physiological and molecular mechanisms mediated by the microbial biostimulant in paddy.

## Materials and methods

### Field experimental design

The field experiment to validate methane-derived microbial biostimulant on yield and GHG emission was conducted for three seasons between 2021 and 2023. The field layout is shown in **Supplementary Figures S1A–C**.

### Field operations and nutrient management

The agroecological conditions during the cropping in Vellore, Tamil Nadu, India, were tropical wet and dry climates, typical of savanna regions. The complete cropping cycle was sunny with moderate rain by a pronounced dry season during the high-sun months, where temperatures were higher, and precipitation levels were lower. Soil nature was clay loam type. The field was carefully prepared for the trials with bunds and buffer channels in place to prevent cross-infiltration. Each plot was allocated a specific irrigation channel to ensure that water applied to one plot remained contained within that plot. This design effectively prevented cross-infiltration. To maintain a uniform plant population, two seedlings were planted per hill with a spacing of 20 cm × 10 cm.

In all the three seasons, recommended nitrogen doses were made as split applications: basal, tillering, and panicle initiation stages. The recommended dose of fertilizers [Nitrogen, phosphorous, and potassium (NPK)] was applied at dose of 100:50:50 kg/ha. The fertilizer was supplied through different sources such as urea (46% N), single super phosphate (16% P_2_O_5_), and muriate of potash (60% K_2_O) as per the package of practice. For treatments with 75% of N, a half dose of N and a full dose of P_2_O_5_ and K_2_O were applied as basal doses. The remaining N was split into two doses at the tillering stage and panicle initiation stage. Crops were monitored carefully and maintained to remain pest-free. Curacron^®^ and Coragen^®^ were applied at 2.5 mL/L and 0.5 mL/L of water, respectively, to control the early stages of leaf folder and stem borer incidents.

### Season I study

The season I study was conducted from June to October 2021 at VIT School of Agricultural Innovations and Advanced Learning, Vellore Institute of Technology, Vellore, Tamil Nadu, India. Plots were prepared in dimensions of 5 m × 5 m. The experimental design followed the randomized block design (RBD) method, with five treatments and three replicates. Treatment details are as follows: control (T1), 10 mL/L dose of methane-derived microbial biostimulant under two different microbial biostimulant application conditions (T2 and T3), 75% nitrogen (N) control (T4), and 75% N + 10 mL/L dose of methane-derived microbial biostimulant application (T5).

### Season II study

The season II study was carried out from February to June 2022 in a farmer’s field near Vellaikal Medu, Vellore, Tamil Nadu, India. The testing area covered an area of 200 m^2^ for each treatment, and the details are as follows: control (T1) and 10 mL/L dose of methane-derived microbial biostimulant (T2). For season I and season II, ASD-16 seeds released by Tamil Nadu Agricultural University, Coimbatore, India, were used for the study.

### Season III study

For season III, the testing covered an area of 800 m^2^ for each treatment. The testing was conducted from July to November 2023. The season III study was carried out in a different farmer’s field near Vellaikal Medu, Vellore, Tamil Nadu, India. Treatment details are as follows: control (T1) and 10 mL/L dose of methane-derived microbial biostimulant (T2). For season III, ADT-37 seeds released by Tamil Nadu Agricultural University, Coimbatore, India, were used for the study.

### Microbial biostimulant application

The methane-derived microbial biostimulant (CleanRise^®^) is manufactured by String Bio, India, using an IP-protected fermentation process. The active ingredient in microbial biostimulant are cells of *Methylococcus capsulatus* derived by an innovative fermentation, downstream processing, and formulation process (PCT application No. WO2021240472A1; whole-cell methanotroph-based biostimulant compositions, methods, and applications thereof). Two different levels of fertilizer application were followed for the season I study. With 100% NPK application and 75% N + 100% P&K as fertilizer input with 10 mL/L of microbial biostimulant. In each of these cases, respective controls with 100% NPK and 75% N + 100% P&K were maintained. For the season II and season III studies, microbial biostimulant at 10 mL/L were evaluated under the 100% NPK level. Three applications of microbial biostimulant at 10 mL/L (corresponding to 15 L/ha considering 500 L as water dilution volume/ha) were performed during the crop growth. Roots of 20-day-old seedlings were immersed in microbial biostimulant solution for 20 min prior to transplantation to the main field. During the season I study, a soil application (for T2) and foliar application (for T3 and T5) during the tillering stage was given as second application. Third application was performed as foliar spray for all treatments (T2, T3 and T5) during panicle development stage. For season II and season III, seedling root dipping during transplantation and foliar application during the tillering stage and panicle development stage were followed for microbial biostimulant treatment. Control plants received water spray at the same time.

### Grain and straw yield measurements

Grain yield in microbial biostimulant-treated plots was compared with the respective control treatments. The harvest index (HI) was computed following the method of Du et al. (2022). The number of productive tillers and grains/spikelets were counted manually from five independent plants from control (T1) and methane-derived microbial biostimulant-treated plants (T3). Thousand grains were taken from panicles of five independent plants from each treatment, and their weight was recorded as test weight (season I study). Test weight was expressed in grams. For the season I study, grain and straw yield from individual treatments were averaged from the three replicated plots and extrapolated the values to get yield per hectare. For the season II and season III studies, bulk harvest from each treatment area was extrapolated to arrive at yield per hectare.

### Physiological parameter measurements in paddy

Physiological parameters were analyzed from five tagged plants from each treatment (T1 and T3) during the season I study. The photosynthetic rate was analyzed using an Infrared gas Analyzer (IRGA, Licor-6800; Li-Cor Inc., Lincoln, NE, USA). Leaf stomatal conductance and transpiration rate were analyzed using a LICOR 6800 portable photosynthesis system (Lincoln, Nebraska, USA). These observations were recorded on clear sunny days between 10:00 a.m. and 12:00 p.m. in a saturated light environment. Physiological parameters were collected 7 days after the first foliar application of microbial biostimulant.

### RNA extraction and quantitative reverse transcriptase–PCR analysis

Total RNA extraction, cDNA synthesis, and quantitative reverse transcriptase–PCR (qRT-PCR) were carried out as described by Kumar et al. (2018). The samples were collected from season I trials (T1 and T3). Emerging leaves and panicles of equal developmental stages from five independent plants were pooled before RNA extraction. RNA from leaves (collected during peak tillering stage) and panicles (collected during panicle initiation stage) from control and treatments were isolated as reported previously (Kumar et al., 2018). Leaf RNA and panicle RNA were normalized using *Osactin*, which was previously reported as an appropriate endogenous gene in rice (Dai et al., 2008). Fold-change differences in gene expression were analyzed using the comparative cycle threshold (*Ct*) method. Relative quantification was carried out by calculating *Ct* to determine the fold difference in gene expression [Δ*Ct* target − Δ*Ct* calibrator]. The relative expression level was determined as 2^−ΔΔCt^. Primers used for the analysis are mentioned in **Supplementary Table S1**.

### CH_4_ and N_2_O emission measurements

The static closed chamber method as mentioned in Minamikawa et al. (2015) was used for gas sample collection in this study. The number and position of chambers are included in **Supplementary Figure S1A–C**, and gas samples were collected from each of the three replicate chambers from each treatment. For the season I study, gas samples were collected at three time points [40, 60, and 80 days after transplantation (DAT)], which correspond to the active tillering stage, panicle initiation stage, and grain filling stage, respectively. For season II and season III, gas samples were collected every 10 days after transplantation to harvest. CH_4_ and N_2_O were analyzed using gas chromatography with flame ionization detection (FID) and electron capture detection (ECD), respectively. CH_4_ and N_2_O fluxes were calculated as reported by Parthasarathi et al. (2019) and Nascimento and Rodrigues (2019) and expressed in g·ha^−1^·h^−1^. The equivalent CO_2_ (CO_2_eq) emission for total CH_4_ and N_2_O was computed as reported previously (Oo et al., 2018).

### Statistical analysis

Average mean, standard error (SE), and number of replicates (n) used for each experiment were employed for statistical analysis using the GraphPad QUICKCALC online software (http://www.graphpad.com/quickcalcs/ttest1.cfm). The statistical significance of differences between controls and samples was tested according to the unpaired Student’s *t*-test.

Additional details about the methodology used in the study that are not detailed here are mentioned in the **Supplementary Methods File**.

## Results

### Methane-derived microbial biostimulant improves grain yield in paddy

**Figure 1:**
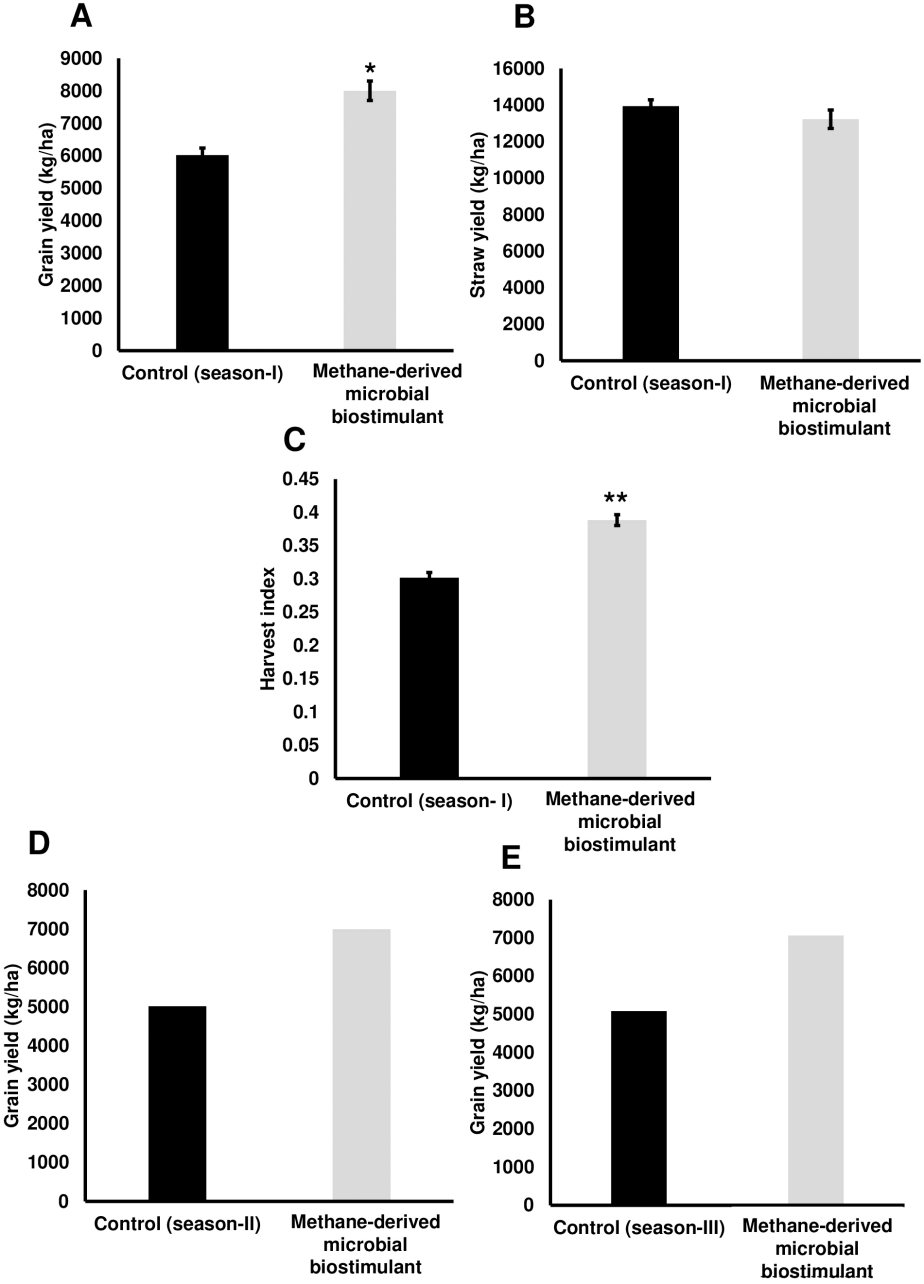
Methane-derived microbial biostimulant increases grain yield and harvest index in rice. **(A)** Effect of microbial biostimulant on grain yield improvement in paddy from season I validation. Methane-derived microbial biostimulant application resulted in 32% improvement in grain yield compared to control. **(B)** Impact of methane-derived microbial biostimulant on straw yield. There was no significant change in levels of straw yield between the treatments. **(C)** Impact of methane-derived microbial biostimulant on harvest index in rice. A significant increase in harvest index of 0.39 was observed in microbial biostimulant-treated plants compared to controls (0.30). **(D, E)** Influence of methane-derived microbial biostimulant on grain improvement in paddy from season II and season III validation. Grain yield improvement of~ 39% was observed during second-season and third-season validation. Control (season I), control (season II), and control (season III) represent the yield observed in control plots at each season. The area covered for the second and third season trials were 200m^2^ and 800m^2^ respectively per treatment and bulk harvest was performed; hence, error bar is not shown in the data. Differences were evaluated using the two-tailed Student’s *t*-test and *P* < 0.05 and *P* < 0.01 are represented by “*” and “**”, respectively.

To evaluate the effect of a methane-derived microbial biostimulant (CleanRise^®^) on rice yield, open-field experiments were conducted across three seasons from 2021 to 2023. During the season I trial, the average grain yield improvement induced by microbial biostimulant varied between 32% and 39% (T3—8,004 ± 299 kg/ha to T2—8,400 ± 80 kg/ha in microbial biostimulant treatment vs. T1—6,024 ± 216 kg/ha in control plots under 100% NPK levels) (**Figure 1A**; **Supplementary Figure S2A**). With the application of microbial biostimulant, a significant increase in the number of grains per spikelet and test weight was also observed (**Table 1**). Although we observed a marginal increase in the number of productive tillers with the application of microbial biostimulant (16.33 ± 1.20 in microbial biostimulant treatment vs. 12.66 ± 0.88 in control plants), there was no statistically significant difference between control and treatments with respect to straw yield (T3—13,119 ± 763 kg/ha to T2—13,230 ± 505 kg/ha in microbial biostimulant treatment vs. T1—13,938 ± 350 kg/ha in control plots under 100% NPK levels) (**Figure 1B**). An informative indicator of the sink–source balance in plants is the HI. While the HI observed for controls was 0.30, the HI for microbial biostimulant-treated plants ranged between 0.38 and 0.39 (**Figure 1C**). During the season II validation, microbial biostimulant application resulted in ~39% improvement in grain yield (6,997 kg/ha in microbial biostimulant treatment vs. 5,015 kg/ha in control plots) (**Figure 1D**). The season III study showed a grain yield improvement of 39% over the control practice (7,058 kg/ha in microbial biostimulant treatment vs. 5,081 kg/ha in control plots) (**Figure 1E**). Multi-location yield trials were performed to assess the stable performance and adaptability of microbial biostimulant over a broad range of environmental conditions. Application of microbial biostimulant resulted in improved yield across different seasons/ecological regions, and the yield varied between 15% and 27% (**Supplementary Figures S2B–D**).

**Table 1:** Grain yield component traits in methane-derived microbial biostimulant-treated paddy.

Treatment	Grains/spikelets	Test weight (g)	Grain yield (kg/ha)	Yield improvement (%)
T1—Control Season I	128 ± 6.7	22 ± 0.86	6024 + 216	0
T3—Microbial biostimulant	166 ± 6.7*	28 ± 1.12*	8004 + 299**	32

Yield related traits mentioned are average data collected from five independent plants from season I study. Differences were evaluated using the two-tailed Student’s t-test, and *P* < 0.05 and *P* < 0.01 are represented by “*” and “**”, respectively.

**Figure 2:**
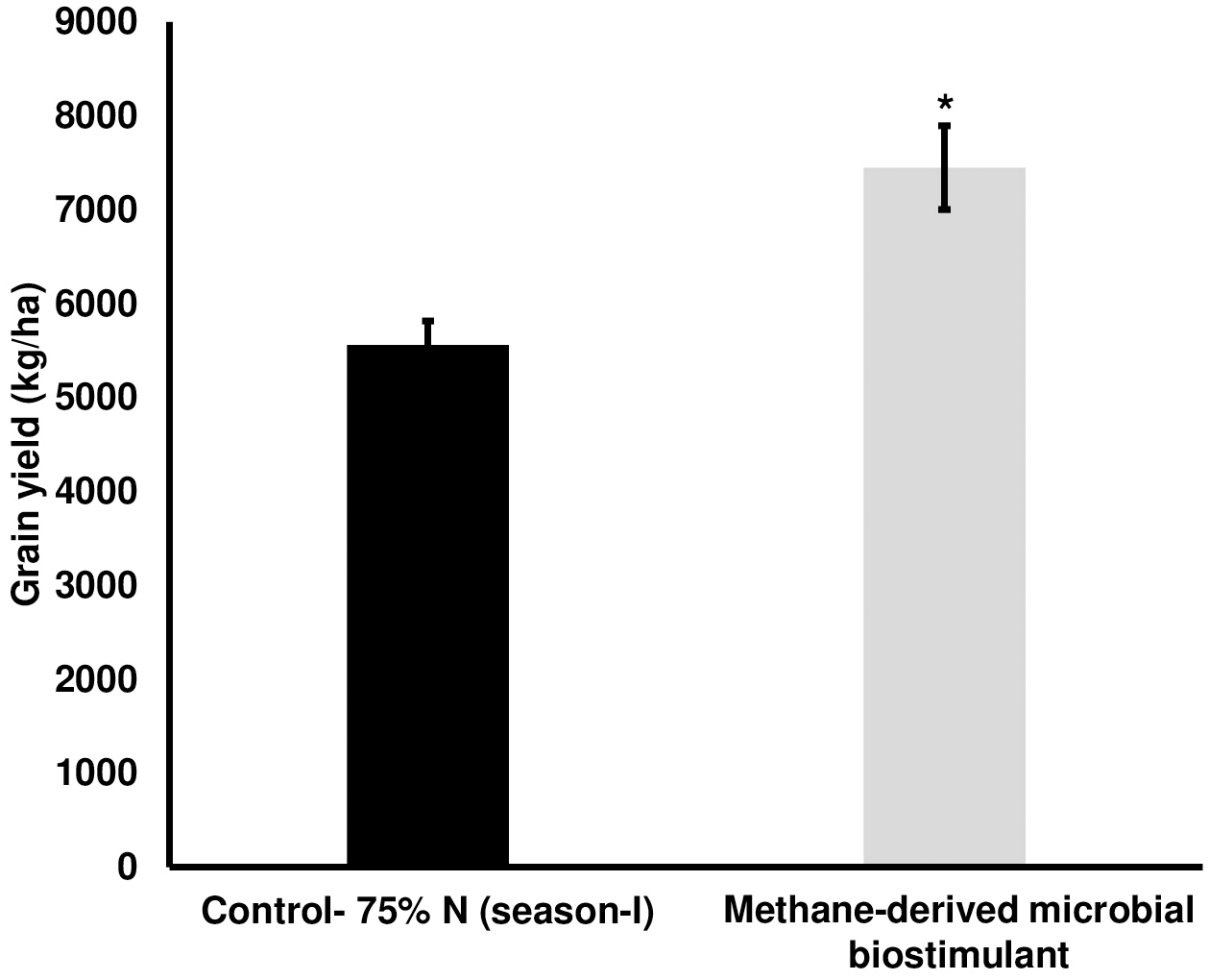
Effect of microbial biostimulant on grain yield improvement in paddy with 75% nitrogen (N) fertilizer. Methane-derived microbial biostimulant application resulted in 34% improvement in grain yield compared to 75% N control. Differences were evaluated using the two-tailed Student’s *t*-test, and significant differences at *P* < 0.05 are represented by *.

We next evaluated the effect of microbial biostimulant on rice under reduced N fertilizer levels. The reduction in fertilizer for the control and treated fields was managed as outlined in the Materials and Methods section. Interestingly, grain yield under reduced N were 5,561 ± 253 kg/ha in the control plot, whereas microbial biostimulant application resulted in 7,448 ± 446 kg/ha (**Figure 2**), resulting in a 34% improvement. Microbial biostimulant application improved root growth compared to control plants (**Supplementary Figures S3A, B**) under different fertilizer levels. Microbial strain in biostimulant produced 1.83–3.61 mg/L indole-3-acetic acid (IAA) in the presence of tryptophan (**Supplementary Table S2**).

### Methane-derived microbial biostimulant regulates photosynthesis, tillering and panicle architecture, and nutrient transport in paddy

**Figure 3:**
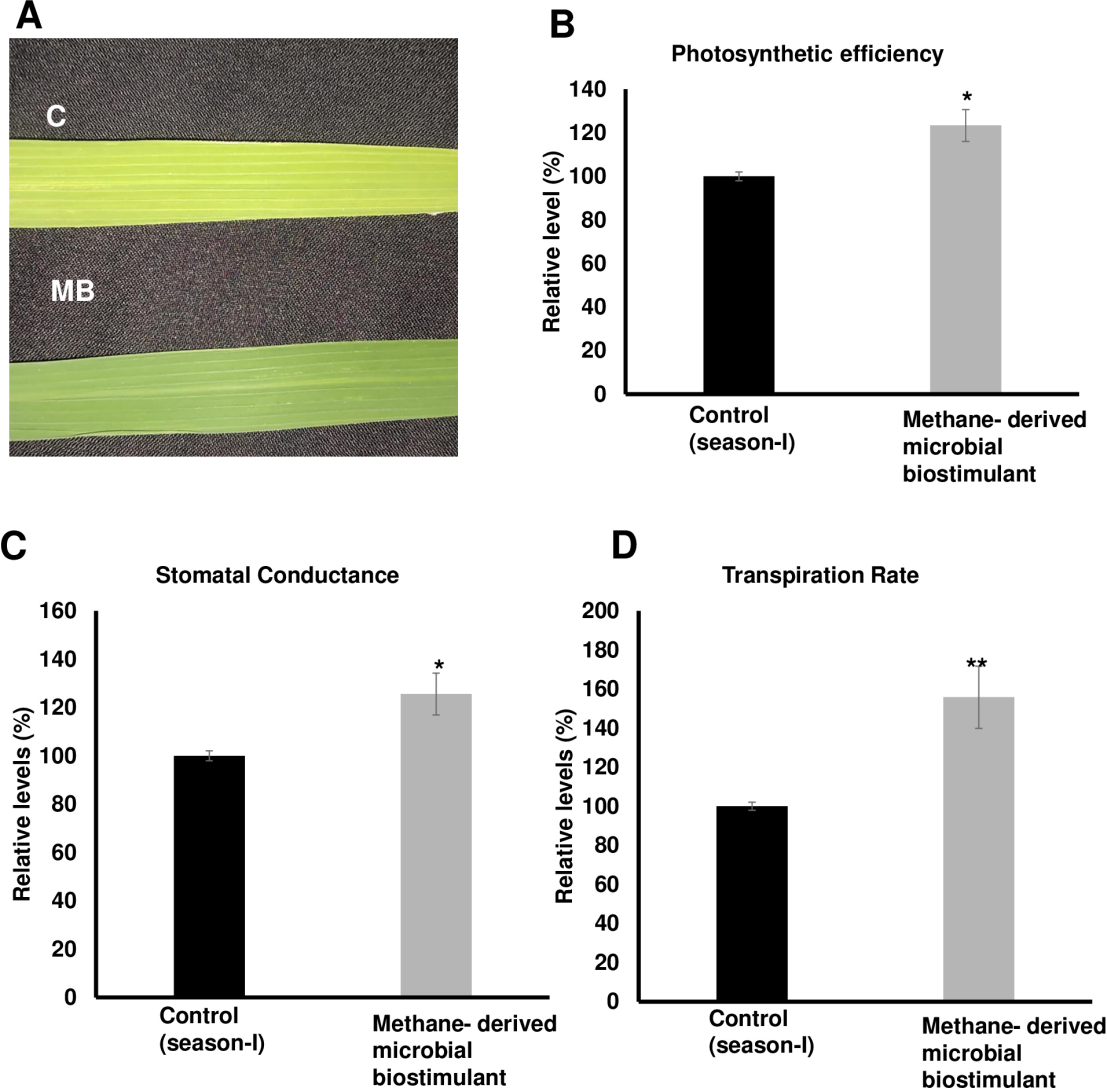
Effect of methane-derived microbial biostimulant on leaf phenotype and physiological traits in paddy leaves. **(A)** Phenotypic feature of microbial biostimulant-treated paddy leaves. Influence of methane-derived microbial biostimulant on greenness in paddy leaf: control leaf **(C)** and methane-derived microbial biostimulant-treated leaf (MB). **(B)** Photosynthetic efficiency, **(C)** stomatal conductance, and **(D)** transpiration rate are represented as % relative to control plants. Student’s *t-*test: significant differences at *P* < 0.05 and *P* < 0.01 are represented by “*” and “**”, respectively.

**Figure 4:**
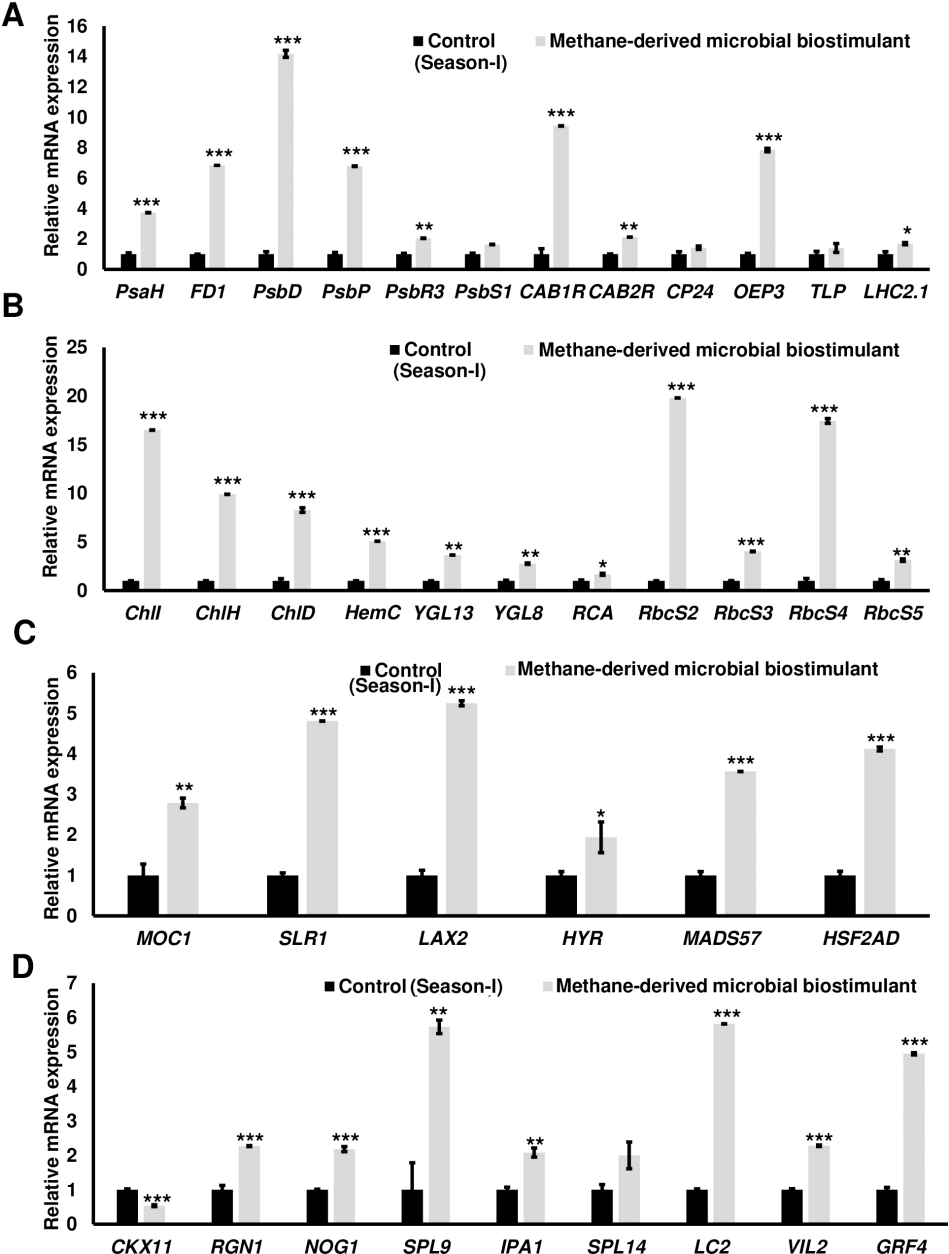
Microbial biostimulant acts as a master regulator of photosynthesis, tillering, and panicle architecture. Reverse transcriptase–quantitative polymerase chain reaction (RT-qPCR) analysis showing the relative expression of genes related to photosynthesis **(A, B)**, tillering **(C)**, and panicle architecture **(D)** in rice with or without microbial biostimulant application. Expression levels of genes were normalized to the endogenous reference gene *actin* and are represented relative to respective controls, which were set to 1. Pooled leaves or panicles from three to five plants were used for RNA extraction. The results shown are from three independent experiments. *PsaH*, Photosystem I reaction center subunit VI; *FD1*, Ferredoxin 1; *PsbD*, Photosystem II D2 protein; *PsbP*, photosystem II subunit P; *PsbR3*, photosystem II subunit PsbR3; *PsbS1*, Photosystem II 22 kDa protein 1; *CAB1R*, Chlorophyll a–b binding protein 1; *CAB2R*, Chlorophyll a–b binding protein 2; *CP24*, Chlorophyll Protein 24; *OEP3*, Oxygen-evolving enhancer protein-3; *TLP*, Thylakoid luminal protein; *LHC2.1*, Chlorophyll a–b binding protein 2.1/Light Harvesting Complex Protein 2.1; *ChlI*, Magnesium-chelatase subunit ChlI; *ChlH*, Magnesium-chelatase subunit ChlH; *ChlD*, Magnesium-chelatase subunit Child; *HemC*, porphobilinogen deaminase/hydroxymethylbilane synthase; *YGL13*, yellow-green leaf 13; *YGL8*, yellow-green leaf 8; *RCA*, Rubisco activase; *RbcS2*, 3, 4, and 5, Ribulose bisphosphate carboxylase small subunit; *MOC1*, monoculm 1; *SLR-1*, Slender Rice-1; *LAX2*, LAX PANICLE2; *HYR*, HIGHER YIELD RICE; *MADS57*, MADS-box transcription factor; *HSF2AD*, Heat Stress Transcription Factor 2D; *CKX11*, Cytokinin oxidase/dehydrogenase; *RGN1*, Regulator of Grain Number-1; *NOG1*, Number of Grains-1; *SPL9* and *SPL14*, SQUAMOSA Promoter Binding Protein-Like; *IPA1*, Ideal Plant Architecture-1; *LC2*, Leaf Inclination 2/VIN3 (vernalization insensitive 3-like protein); *VIL2*, VIN3-LIKE 2 protein; *GRF4*, Growth Regulating Factor 4. Differences were evaluated using the two-tailed Student’s *t*-test, and significant differences at *P* < 0.05, *P* < 0.01, and *P* < 0.001 are represented by “*”, “**”, and “***”, respectively.

The microbial biostimulant-treated paddy fields had visible differences in field lushness and showed brilliant dark-green leaves compared to control fields (**Figure 3A**). Physiological analysis showed that microbial biostimulant application resulted in an 18% increase in photosynthetic rate, a 22% increase in stomatal conductance, and an ~48% increase in transpiration rate (**Figures 3B–D**). To elucidate the molecular mechanism affecting the phenotype in microbial biostimulant-applied plants, mRNA expression analysis of genes encoding enzymes involved in photosynthesis, tillering, and panicle architecture was performed. The transcript levels in microbial biostimulant-treated leaves or panicles were compared with respect to control samples. Most of the genes related to photosynthesis were upregulated between 1.4- and ~20-fold in plants applied with microbial biostimulant. The upregulated genes were related to all major components of photosynthesis, including, Photosystem I [Photosynthetic reaction center I protein family (*PsaH*); Photosystem I–Ferredoxin-1 (*FD1*)], Photosystem II [Photosynthetic reaction center II protein family (*PsbD*, *PsbP*, *PsbR3*, and *PsbS1*), Chlorophyll A/B binding protein (*CAB*) genes (*CAB1R*, *CAB2R*, and *CP24*); oxygen-evolving enhancer protein-3 (*OEP3*); Light Harvesting Complex II (*LHC2.1*); thylakoid lumenal 19 kDa protein chloroplast precursor (*TLP*)], chlorophyll biosynthesis pathway [Magnesium-Chelatase subunits (*CHLI*, *CHLH*, and *CHLD*); hemC gene encoding porphobilinogen deaminase (*HEMC*); Yellow-Green Leaf (*YGL13* and *YGL8*)], and enzymes involved in the CBB (Calvin–Benson–Bassham) cycle [Ribulose-1,5-Bisphosphate carboxylase/oxygenase Rubisco activase (*RCA*); Rubisco small subunit gene (*RbcS2*, *RbcS3*, *RbcS4*, and *RbcS5*)] (**Figures 4A, B**).

To understand the molecular mechanism of enhanced photosynthetic capacity on axillary meristem growth and panicle architecture, the transcript levels of critical genes involved in the regulation of shoot branching, panicle, and grain development were examined. Upon microbial biostimulant application, a 1.9- to 5.2-fold increase was observed in the expression of tillering-related genes like monoculm 1 (*MOC1*), Slender Rice-1 (*SLR1*), Lax panicle (*LAX2*), Higher Yield Rice (*HYR*), MADS-box transcription factor (*MADS57*), and Heat Stress Transcription Factor 2D (*HSF2AD*) (**Figure 4C**). As photosynthate partitioning from the source (leaf) to the sink (grains) is critical for panicle development and grain filling, mRNA expression of key genes including Cytokinin oxidase/dehydrogenases (*CKX11*), Regulator of Grain Number-1 (*RGN1*), Number of Grains-1 (*NOG1*), SQUAMOSA Promoter Binding Protein-Like (*SPL9* and *SPL14*), Ideal Plant Architecture-1 (*IPA1*), Leaf Inclination 2 (*LC2*), Vernalization insensitive 3-like protein (*VIL2*), and Growth-Regulating Factor 4 (*GRF4*) involved in grain development was further analyzed. A 2- to 5.7-fold upregulation of genes controlling panicle architecture was observed in microbial biostimulant-treated samples, indicating that improved photosynthetic capacity positively translated to grain filling and development (**Figure 4D**). Interestingly, microbial biostimulant application also downregulated the expression of *CKX11*, a negative regulator of panicle architecture in paddy.

To understand the impact of microbial biostimulant on nutrient uptake, the expression of key genes encoding macronutrient transporters was monitored. RT-qPCR analyses showed that the genes involved in nitrogen uptake and transport were overexpressed by 2- to 12-fold in microbial biostimulant-treated paddy roots over control (**Supplementary Figure S4A**). High-affinity potassium, phosphate, and zinc transporters were also upregulated ranging from 2- to 10-fold in microbial biostimulant-treated roots over control (**Supplementary Figure S4B**).

### GHG mitigation potential of methane-derived microbial biostimulant

**Figure 5:**
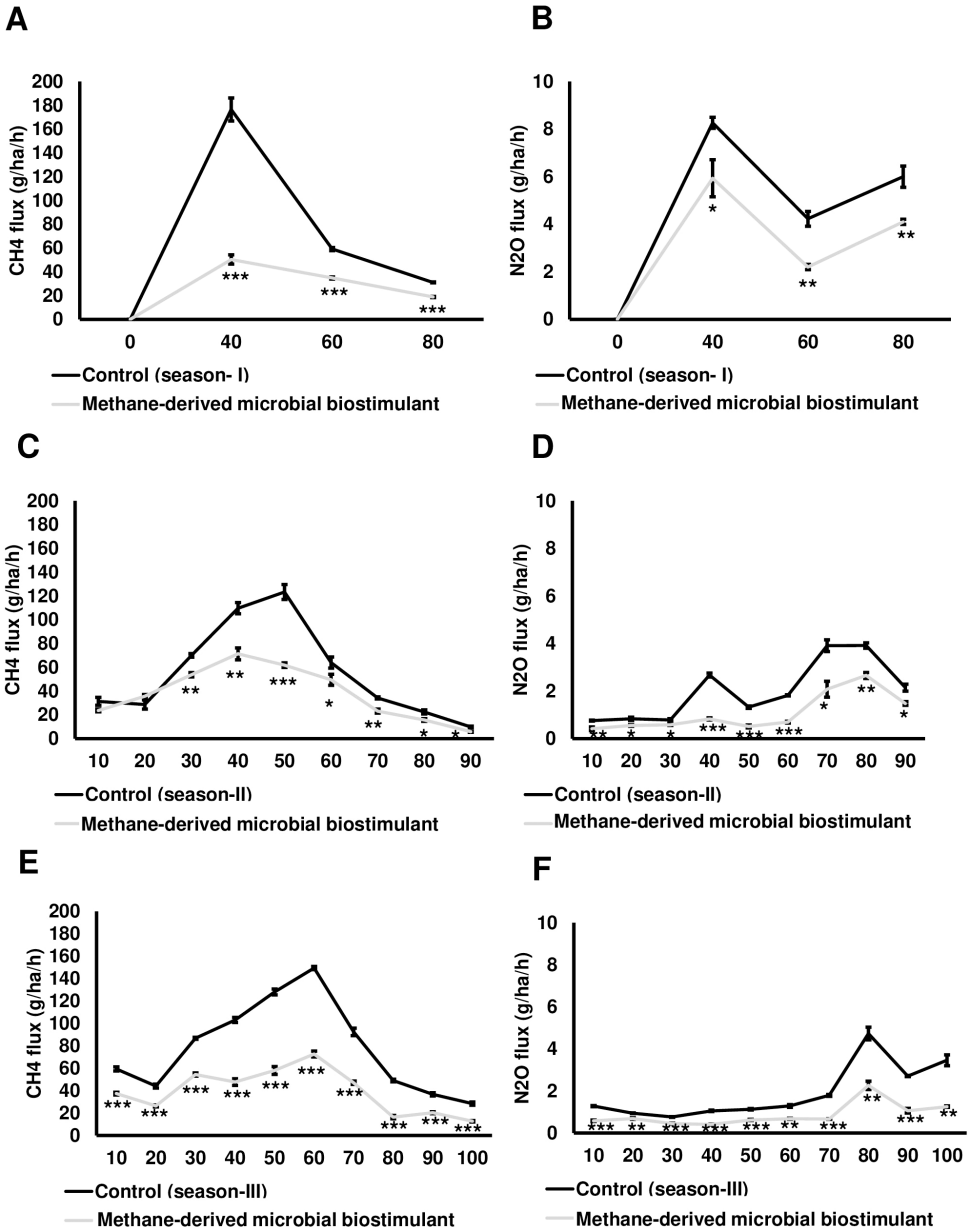
Greenhouse gas mitigation mediated by methane-derived microbial biostimulant. Influence of microbial biostimulant on CH_4_ and N_2_O emission from rice field. Gas samples were collected in triplicate from each plot for every time point and analyzed using gas chromatography with flame ionization detection (FID) and electron capture detection (ECD). While gas samples were collected at three time points for season I trial, gases were collected every 10 days during season II and season III trials. CH_4_ and N_2_O flux were calculated and expressed as g·ha^−1^·h^−1^. Microbial biostimulant application resulted in ~60% reduction in CH_4_ **(A)** emission from rice fields, whereas there was 34% reduction in N_2_O **(B)** during the course of plant growth of season I study. Methane-derived microbial biostimulant use resulted in ~31% reduction in CH_4_ **(C)** emission from rice fields, whereas there was ~47% reduction in N_2_O **(D)** during season II validation. During season III validation, methane-derived microbial biostimulant use resulted in a 48% reduction in CH_4_ **(E)** emission from rice fields, whereas it was ~50% reduction in N_2_O **(F)**. Control (season I), control (season II), and control (season III) represent the emission observed in control plots from season I, season II, and season III validation, respectively. Differences were evaluated using the two-tailed Student’s *t*-test, and *P* < 0.05, *P* < 0.01, and *P* < 0.001 are represented by “*”, “**”, and “***”, respectively.

In this study, we also monitored the effect of microbial biostimulant on the flux of CH_4_ and N_2_O from rice paddies during three time points of crop growth (40, 60, and 80 DAT) of the season I study. The dynamic fluxes of CH_4_ and N_2_O over the rice growing period were strongly affected by the microbial biostimulant application. In our study, CH_4_ flux was high during the tillering stage and then gradually decreased toward the panicle initiation stage and end of the growing period across all the three seasons. CH_4_ emission varied considerably among the treatments, and the dynamics of CH_4_ flux during the cropping seasons is presented in **Figure 5A**. Microbial biostimulant application resulted in a reduction of approximately 70% in CH_4_ emissions at 40 DAT (46.43 ± 3.78 g·ha^−1^·h^−1^ CH_4_ in microbial biostimulant-treated plots vs. 176.50 ± 9.65 g·ha^−1^·h^−1^ CH_4_ in control plots). Approximately 50% reduction in emission was recorded during subsequent sampling at 60 DAT (29.30 ± 1.58 g·ha^−1^·h^−1^ CH_4_ in microbial biostimulant-treated plots vs. 59.20 ± 1.30 g·ha^−1^·h^−1^ in control plots) and 80 DAT (14 ± 1.30 g·ha^−1^·h^−1^ CH_4_ in microbial biostimulant-treated plots vs. 31.36 ± 0.31 g·ha^−1^·h^−1^ in control plots). Although the levels of N_2_O emissions were much lower compared to CH_4_ flux, a similar emission pattern was observed. Fluxes of N_2_O at the farms varied from 2.30 g·ha^−1^·h^−1^ to 5.76 g·ha^−1^·h^−1^ in microbial biostimulant treatment compared to 4.23 g·ha^−1^·h^−1^ to 8.26 g·ha^−1^·h^−1^ in control plots during the cropping season (**Figure 5B**). The highest N_2_O flux of 8.26 ± 0.23 g·ha^−1^·h^−1^ was recorded during early crop growth in control plants. Here, microbial biostimulant application led to a significant reduction in N_2_O emission of up to 30% (5.76 ± 0.29 g·ha^−1^·h^−1^). Microbial biostimulant-mediated reduction in N_2_O flux was ~45% during the second (2.30 ± 0.05 g·ha^−1^·h^−1^ in microbial biostimulant-treated plots vs. 4.23 ± 0.31 g·ha^−1^·h^−1^ in control plots) and third (3.93 ± 0.31 g·ha^−1^·h^−1^ in microbial biostimulant-treated plots vs. 6.0 ± 0.45 g·ha^−1^·h^−1^ in control plots) sampling periods.

During season II trials, we sampled at greater frequency during the crop growth stage. While no significant change in CH_4_ emission levels was observed at 10 and 20 DAT, we recorded a drastic reduction ranging from 23% to 50% during the subsequent sampling period (**Figure 5C**; **Supplementary Table S3A**). N_2_O reduction during season II varied between 30% and 70% during the crop growth (**Figure 5D**; **Supplementary Table S3A**). Our season III validation (GHG sampled at a 10-day frequency during the crop growth stage) also showed a significant reduction in CH_4_ and N_2_O flux in plots applied with microbial biostimulant. We observed a 37%–67% reduction in CH_4_ and a 27%–64% reduction in N_2_O flux in microbial biostimulant-applied paddy plots compared to control plots in season III trials (**Figures 5E, F**; **Supplementary Table S3A**). The average CH_4_ and N_2_O emissions for all three cropping seasons during the entire crop cycle between control and microbial biostimulant-treated plots are shown in **Supplementary Table S3B**. Taken together, from three-season trials, the cumulative CH_4_ and N_2_O emissions were 31%–61% and 34%–50%, respectively, lower compared to those of control fields (**Supplementary Table S4**).

### Impact on yield-scaled CO_2_ reduction mediated by methane-derived microbial biostimulant

In the present study, the contribution of CH_4_ to the total global warming potential (GWP) ranged from ~11,045 to 19,065 kg CO_2_/ha/season during three different seasons in the control field, whereas it was 6,924–9,897 kg CO_2_/ha/season in microbial biostimulant-treated fields. Yield-scaled CO_2_ equivalent of CH_4_ emission from controls ranged from 2,204 to 3,752 kg CO_2_-eq/t, whereas it ranged from 865 to 1,402 kg CO_2_-eq/t with microbial biostimulant application (**Supplementary Table S4**). Similarly, N_2_O equivalent CO_2_ emission from fields with microbial biostimulant application was 459–3,197 kg CO_2_/ha/season compared to 923–4,839 kg CO_2_/ha/season in control fields. Yield-scaled CO_2_ equivalent of N_2_O emission from control fields ranged from 182 to 803 kg CO_2_-eq/t and 65–399 kg CO_2_-eq/t with microbial biostimulant application (**Supplementary Table S4**).

## Discussion

The demand for increased agricultural production in the context of limited arable land and climate change necessitates innovative solutions for sustainable agricultural practices. Global rice consumption has increased markedly over the last several decades, and rice demand is projected to increase by 28% by 2050. Nevertheless, rice yields have stagnated in 35% of all rice-growing regions (Ray et al., 2012). As the world’s population continues to grow at an alarming rate, an important challenge for future rice cultivation is to increase crop yield substantially while simultaneously reducing GHG emissions. The application of microbial biostimulants has recently gained significant attention for enhancing yield potential in plants (Castiglione et al., 2021; Hijri, 2023; Kaushal et al., 2023). Microbial partners have been shown to colonize the plant, increase the supply of nutrients to the host, and affect plant performance by producing a variety of metabolites, including phytohormones. Among the different classes of microbial biostimulants, methanotrophs based solutions are less explored for their plant growth-promoting properties. Aerobic methanotrophs are important habitats of rice fields, commonly found in the soil rhizosphere and endosphere of rice plants. While most of the strains can fix atmospheric nitrogen, some of the methanotrophs are also reported to possess phosphate solubilization, potassium, and zinc mobilization activities (Rani et al., 2021a). The effect of seed treatment or foliar application of methanotrophic consortia has been validated previously in small-scale plots (Jeya Bharathi et al., 2018; Taopan et al., 2018; Davamani et al., 2020; Rani et al., 2021b; Mohite et al., 2023). For instance, methanotrophic consortia application did not alter the grain yield or straw yield under 100% fertilizer conditions; however, a 25% increase in grain yield without altering straw yield was observed with methanotrophic consortia treatment at 50% N levels (Taopan et al., 2018). Similarly, co-inoculation of *Methylobacterium oryzae* MNL7 and *Paenibacillus polymyxa* MaAL70 in rice improved grain yield by 14% when N was supplied through urea (Rani et al., 2021b). In another study, Mohite et al. (2023) demonstrated the impact of different methanotrophic strains on grain yield under pot conditions. In the present study, through multi-location and three-season trials, we demonstrate an increase in grain yield ranging from 15% to 39% (**Figures 1A, D, E**; **Supplementary Figure S2**) with the application of methane-derived microbial biostimulant in field conditions. The increase in grain yield in microbial biostimulant-treated plots was through a marginal increase in the number of productive tillers with a significant increase in the number of grains per panicle and test weight. Similar to the previous report of Taopan et al. (2018), methane-derived microbial biostimulant did not alter the straw yield in paddy in the present study. Interestingly, methane-derived microbial biostimulant application resulted in significantly improved root growth at 40 and 60 DAT contributing to better NUE (nutrient use efficiency) (**Supplementary Figures S3A, B**). Biostimulant-mediated upregulation of nutrient transporters and modulation of genes related to photosynthetic capacity possibly translated into higher grains per panicle and test weight translating to superior rice yield (**Table 1**, **Figures 3**, **4**; **Supplementary Figure S4**). The positive effect of photosynthates allocation to roots in yield improvement and CH_4_ emission reduction has been reported by Chen et al. (2021).

The combination of high-yielding crop varieties and the widespread use of inorganic fertilizers has markedly improved crop production. However, excessive N input can lead to severe environmental pollution. Methanotrophs are estimated to fix approximately 40 kg N/ha in rice fields and play a crucial role in atmospheric nitrogen fixation (Cao et al., 2022). In this study, even with a 25% reduced N application, the yield per hectare was enhanced in microbial biostimulant-treated plots over the control (75% N) treatment. This could be possibly due to the improved N uptake and translocation influenced by the application of microbial biostimulant. While the optimal requirement of N may vary with soil condition and crop management, the study demonstrates the use of the methane-derived biostimulant as a solution to reduce the N fertilizer to agriculture without impacting farmers’ income.

**Figure 6:**
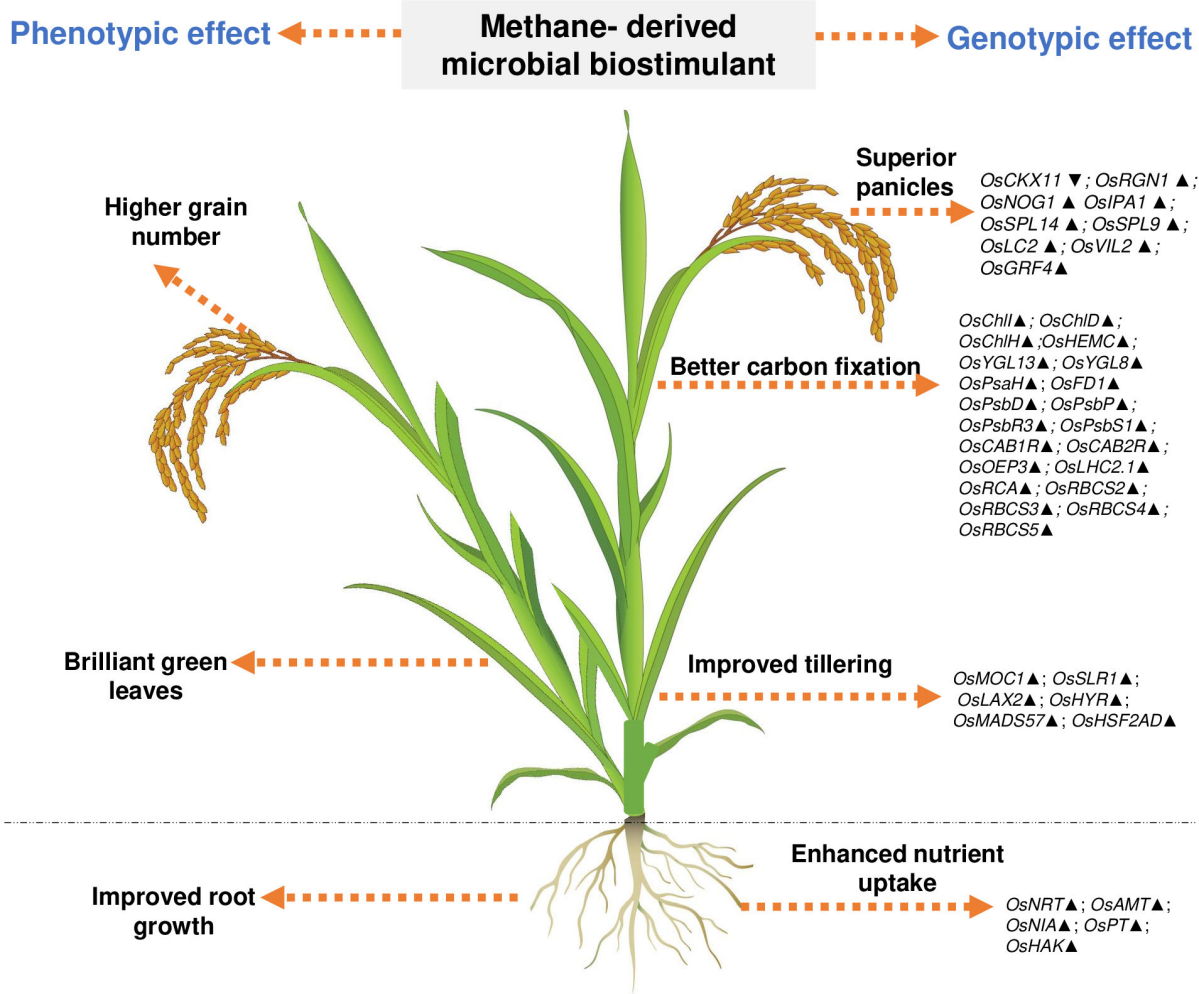
Representative image showing overview of phenotypic and genotypic traits modulated by microbial biostimulant application in rice (*Oryza sativa*). Microbial cells in the biostimulant formulation improve macronutrient availability and transport. Microbial biostimulant application modulates expression of genes involved in axillary bud formation, resulting in more productive tillers. Targeted activation of genes related to chlorophyll biosynthesis pathway and chloroplast development, Photosystem I, Photosystem II, and CBB cycle (Calvin–Benson–Bassham) results in improved carbon fixation. Active photosynthate translocation to developing grain and biostimulant-mediated activation of genes involved in panicle architecture results in a greater number of grains per panicle translating to superior yield. Os, *Oryza sativa*; *NRT*, Nitrate transporter; *AMT*, Ammonium transporter; *NIA*, Nitrate reductase; *PT*, Phosphate transporter; *HAK*, High-affinity potassium transporter; *PsaH*, Photosystem I reaction center subunit VI; *FD1*, Ferredoxin 1; *PsbD*, Photosystem II D2 protein; *PsbP*, photosystem II subunit P; *PsbR3*, photosystem II subunit PsbR3; *PsbS1*, Photosystem II 22 kDa protein 1; *CAB1R*, Chlorophyll a–b binding protein 1; *CAB2R*, Chlorophyll a–b binding protein 2; *CP24*, Chlorophyll Protein 24; *OEP3*, Oxygen-evolving enhancer protein-3; *TLP*, Thylakoid luminal protein; *LHC2.1*, Chlorophyll a–b binding protein 2.1/Light Harvesting Complex Protein 2.1; *ChlI*, Magnesium-chelatase subunit ChlI; *ChlH*, Magnesium-chelatase subunit ChlH; *ChlD* Magnesium-chelatase subunit ChlD; *HemC*, porphobilinogen deaminase/hydroxymethylbilane synthase; *YGL13*, yellow-green leaf 13; *YGL8*, yellow-green leaf 8; *RCA*, Rubisco activase; *RbcS*, Ribulose bisphosphate carboxylase small subunit; *MOC1*, monoculm 1; *SLR-1*, Slender Rice-1; *LAX2*, LAX PANICLE2; *HYR*, HIGHER YIELD RICE; *MADS57*, MADS-box transcription factor; *HSF2AD*, Heat Stress Transcription Factor 2D; *CKX11*, Cytokinin oxidase/dehydrogenase; *RGN1*, Regulator of Grain Number-1; *NOG1*, Number of Grains-1; *SPL9* and *SPL14*, SQUAMOSA Promoter Binding Protein-Like; *IPA1*, Ideal Plant Architecture-1; *LC2*, Leaf Inclination 2/VIN3 (vernalization insensitive 3-like protein); *VIL2*, VIN3-LIKE 2 protein; *GRF4*, Growth Regulating Factor 4. Upward arrow (*▴*) indicates gene upregulation of more than 1.5-fold, and downward arrow (*▼*) indicates more than 50% downregulation of genes.

Understanding the effect of methanotrophs on paddy fields has been studied previously (Jeya Bharathi et al., 2018; Taopan et al., 2018; Davamani et al., 2020; Rani et al., 2021b; Mohite et al., 2023); however, these reports neither have demonstrated insights to the mode of action at the molecular level nor have evaluated the consistency and robustness of the effect. Here, we observe that *M. capsulatus* in the microbial biostimulant formulation was associated with root and leaf tissues of paddy (**Supplementary Figure S5**). This association could have possibly induced a significant effect on host transcriptional regulation. In this study, a comprehensive transcript analysis has been conducted, and the data indicate that the microbial biostimulant could serve as a key regulator of systemic pathways, leading to enhanced photosynthesis, increased productive tiller and grain numbers per panicle, and improved test weight, resulting in superior yield. Based on the phenotypic and genotypic observations, we identified three major routes for the mode of action of microbial biostimulant in rice. First, microbial biostimulant positively regulated multiple genes related to macronutrient uptake and transport, resulting in better NUE (**Supplementary Figures S4**, **S6**). Second, microbial cells were able to produce IAA, thus accelerating auxin-mediated root growth (**Supplementary Table S2**, **Supplementary Figure S3**). Lastly, microbial biostimulant simultaneously regulated pathways regulating photosynthesis, tillering, and panicle development (**Figures 4**, **6**). All the analyzed genes have been reported previously to be crucial for photosynthesis [*PsaH* (Li et al., 2015); *FD1*, *PsbR3*, and *CP24* (Perveen et al., 2020); *PsbD* (He et al., 2018); *PsbP* (Li et al., 2015); *PsbS1* (Fu et al., 2020); *CAB1R* and *CAB2R* (Zhu et al., 2015); *OEP3* and *TLP* (Ambavaram et al., 2014); *LHC2.1* (Umate, 2010); *CHLI*, *CHLH*, and *CHLD* (Zhang et al., 2015); *HEMC* (Liu et al., 2023); *YGL13* (Li S Z et al., 2018); *YGL8* (Zhu et al., 2016); and *RCA, RbcS2*, *RbcS3*, *RbcS4*, and *RbcS5* (Kudo et al., 2020)], tillering [*MOC1* (Li et al., 2003), *SLR1* (Liao et al., 2019), *LAX2* (Tabuchi et al., 2011), *HYR* (Ambavaram et al., 2014), *MADS57* (Guo et al., 2013), and *HSF2AD* (Zhang et al., 2018)], and panicle growth [*CKX11* (Zhang et al., 2021), *RGN1* (Li et al., 2021), *NOG1* (Huo et al., 2017), *SPL9* (Hu et al., 2021), *SPL14* and *IPA1* (Miura et al., 2010), *LC2* (Zhao et al., 2010), *VIL2* (Yang et al., 2012), and *GRF4* (Li S et al., 2018)]. Often, there are several checkpoints to regulate photosynthesis and carbon partitioning in plants (Paul and Foyer, 2001). We propose that the microbial biostimulant mediates transcript modulation along with superior photosynthetic activity, which in turn leads to improved carbon fixation and axillary bud initiation. Further, effective photosynthate partitioning to sink tissues such as panicles and developing grains could have translated to better grain yield. Our results align well with the reports of efficient translocation of carbohydrates from source to sink leading to improved grain yield in paddy (Ambavaram et al., 2014). Previous reports suggest that even a minor increase in net photosynthetic activity translates to better yield in wheat and rice (Parry et al., 2011; Li et al., 2020). It is interesting to note that the methane-derived microbial biostimulant enhanced the expression of positive regulators and downregulated negative regulators in paddy, resulting in improved crop physiological parameters translating into superior yield (**Figures 3**, **4**, **6**). Taken together, our findings systematically highlight the in-depth molecular mechanisms mediated by the microbial biostimulant, involving modulation of critical physiological events such as photosynthesis, tillering, and panicle formation, in rice (**Figures 4**, **6**).

Primarily, rice plants serve as the major conduits for the transfer of CH_4_ from the soil to the atmosphere. Well-developed aerenchyma cells in rice plants make a good passage for the gas exchange between the atmosphere and the soil (Nouchi et al., 1990; Nouchi and Mariko, 1993). The majority of CH_4_ (~90%) formed in rice soil is emitted through aerenchyma in rice plants by the process of diffusion (Bhattacharyya et al., 2019). Although some of the previous studies demonstrated mitigation of CH_4_ emissions from paddy fields, the gas samples either were collected only at very few time points during the crop growth (Jeya Bharathi et al., 2018; Davamani et al., 2020) or the cumulative emission reduction achieved was very low at ~12% (Rani et al., 2021b). Further, most of these studies were carried out on small scales. Our field experiments demonstrate significantly reduced CH_4_ and N_2_O emissions with microbial biostimulant treatment across three seasons (**Figure 5**). A previous report demonstrated that type I methanotrophs have strong CH_4_ biotransformation potential in paddy fields (Zheng et al., 2024). Ecosystems like paddy fields typically contain 500 ppm CH_4_, and the ability of type 1 methanotrophs to utilize such low levels of CH_4_ has been reported recently (He et al., 2023). *M. capsulatus* cells in the biostimulant formulation are thus possibly acting as crucial biological filters to alleviate CH_4_ emissions from paddy fields. With the observed symbiotic association in plants (**Supplementary Figure S5**), it is highly plausible that the cells utilize the methane for its metabolic activity as a source of carbon and energy for their growth and, in turn, benefit the plants in multiple ways. Also, rice paddies utilize one-seventh of N fertilizer, making a more potent zone of N_2_O formation and emissions (Timilsina et al., 2020). Qian et al. (2023) recently reported that there is no general pattern of N_2_O emission; however, the emission peaks can be found after N fertilization events or during draining periods. We have also observed peak emission after the top dressing of fertilizers and during the draining periods. The observed reduction in N_2_O emission from rice could be attributed to improved NUE mediated by cells in the biostimulant formulation (**Supplementary Figure S6**).

**Figure 7:**
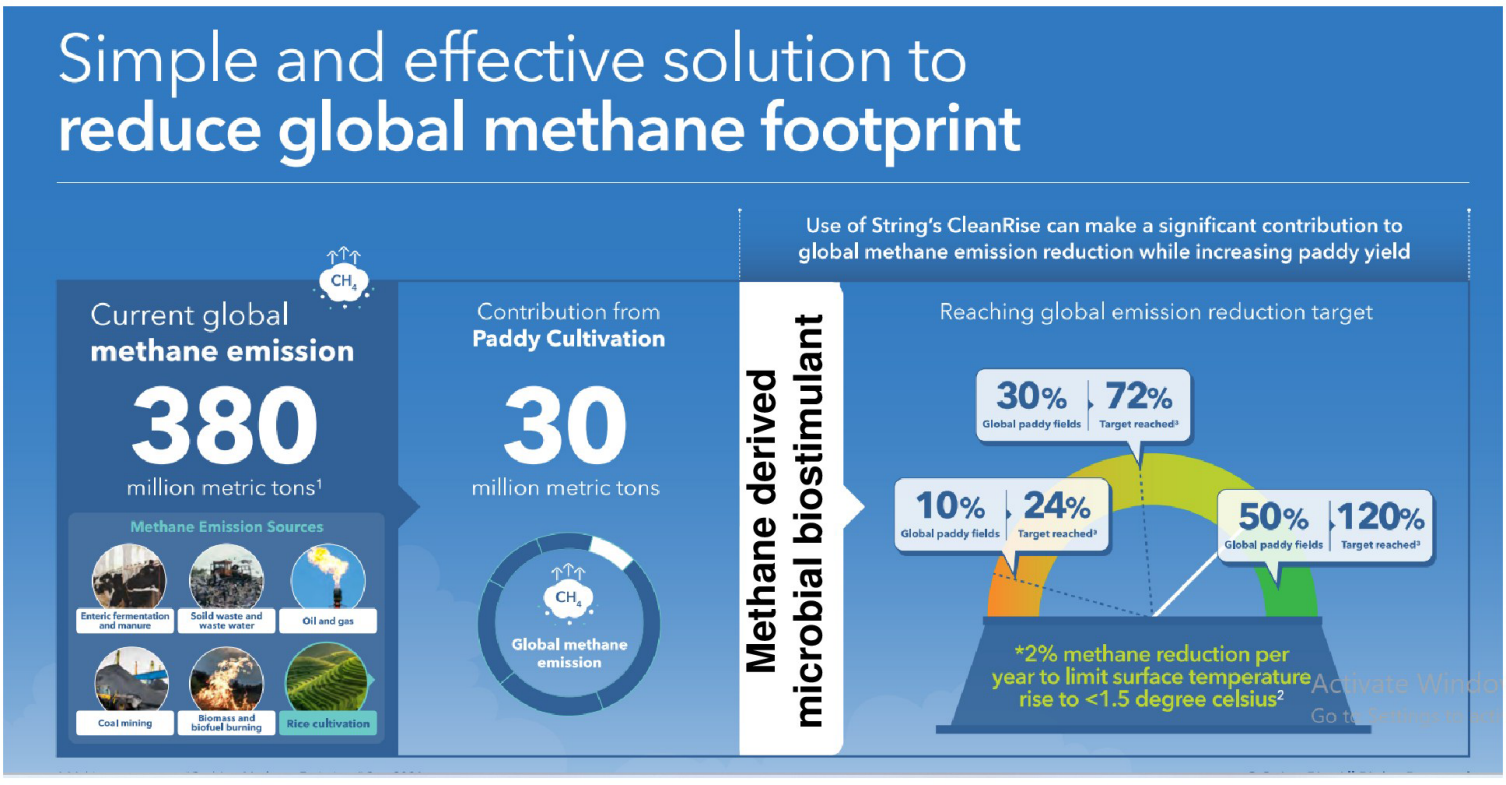
A novel approach to meet COP26’s target for methane reduction by 2030. Globally, rice cultivation contributes to 10% of CH_4_ emission, i.e., 30 million metric tons/year from 162 million hectares of paddy cultivation area. Based on the data from this study, microbial biostimulant application could help to achieve 50% reduction in CH_4_ (a conservative number used for calculation). Using methane-derived microbial biostimulant in 10% of paddy cultivation areas globally, we will be able to reduce methane emissions equivalent to 24% of the proposed target. Further increasing the coverage to 30% of the rice cultivation area could result in 72% of the proposed target. Ambitiously, converting 50% of global rice cultivation to using microbial biostimulant could result in 120% of the proposed target numbers. Note: COP26 targets are 30% reduction in methane emission by 2030 and 50% reduction by 2050 (United Nations Environment Programme/Climate and Clean Air Coalition, 2022; IPCC, 2021).

Although rice is the main staple food for nearly half the world’s population, rice cultivation contributes to an average of 283 kg/ha and 1.7 kg/ha, respectively, to CH_4_ and N_2_O emissions annually (Qian et al., 2023). Rice-growing economies are also among the leading methane emitters globally. For instance, countries such as China, India, and Indonesia have the largest rice cultivation area and contribute to 22%–38%, 11%–19%, and 7%–9% of the 24–37 Tg/year global total, respectively (Crippa et al., 2022). To meet the net zero targets, an ideal goal for different nations now is to reduce short- and long-term emissions without compromising crop yield. Currently, only one-fifth of countries (25/148) mention rice mitigation measures in nationally determined contributions to the Paris Agreement (Rose et al., 2021). Reducing CH_4_ emissions will have a rapid and significant effect on achieving the COP26 target. Here, we offer science-based solutions to prioritize actions aimed at reducing agricultural CH_4_ emissions. At the COP26 meeting, countries aligned on a 2% reduction target in CH_4_ annually, and the data outlined here highlight a powerful path to help achieve these targets. For instance, methane-derived microbial biostimulant application to just 10% of the global paddy-cultivation area (16.2 million hectares) could deliver up to 24% of the global CH_4_ reduction target. Use in 30% of paddy cultivation area (48.6 million hectares) could help in achieving 72% of the global CH_4_ emission target. More ambitiously, enabling use in 50% of the world’s paddy cultivated area (81 million hectares) could deliver 120% of the reduction target (**Figure 7**; **Supplementary Table S5**). The use of a single disruptive solution, such as methane-derived microbial biostimulant, thus could form a promising strategy to curb global CH_4_ emissions from farmed rice without compromising farmers’ welfare while continuing conventional cultivation practices.

## Concluding remarks

Without swift action, emissions in agriculture will continue to increase and contribute to dangerously warming the Earth. We are already experiencing the impacts of climate change with significantly altered climatic patterns. The adoption of sustainable practices is crucial in attaining lower emissions and mitigating the environmental impact. To meet our global commitments to end world hunger by 2030, we need to accelerate the transformation to greener, more resilient, efficient, and sustainable agri-food systems. Here, we demonstrated the potential of methanotrophs to provide benefits for both food security and the environment. Our study shows that the use of methane-derived microbial biostimulant is a win–win–win solution to improve yield, optimize NUE, and reduce GHG emissions from rice fields. It provides the necessary tool to achieve the intensification required to address food security for a growing world population without compromising environmental and climate change mitigation strategies. The mitigation potential highlighted in this study can be realized through targeted policies aimed at catalyzing sustainable rice cultivation globally.

## Data Availability

The original contributions presented in the study are included in the article/**Supplementary Material**. Further inquiries can be directed to the corresponding authors.
