# Supplementary material for: Methane-derived microbial biostimulant reduces greenhouse gas emissions and improves rice yield
Source: Front Plant Sci. 2024 Sep 5;15:1432460. doi: 10.3389/fpls.2024.1432460 (PMC11410644; doi:10.3389/fpls.2024.1432460)
Supplement: Supplementary file 1 [file DataSheet1.pdf]

Methane-derived microbial biostimulant reduces greenhouse gas emission and improves rice yield

Sarma Rajeev Kumar[1, 2] †, Einstein Mariya David[3,4]†, Gangigere Jagadish Pavithra[1], Gopalakrishnan Sajith Kumar [3,4], Kuppan Lesharadevi[3,4], Selvaraj Akshaya[1,2],Chavadi Bassavaraddi[1], Gopal Navyashree[1], Panakanahalli Shivaramu Arpitha[1], Padmanabhan Sreedevi[1,2], Khan Zainuddin[1], Saiyyeda Firdous [3], Bondalakunta Ravindra Babu[1], Muralidhar Udagatti Prashanth[1], Ganesan Ravikumar[1], Palabhanvi Basavaraj[1], Sandeep Kumar Chavana [1], Vinod Munisanjeeviah Lakshmi Devi Kumar[1,2], Theivasigamani Parthasarathi[3] and Ezhilkani Subbian[1,2]*

†- These authors contributed equally to this work

1- String Bio Private Limited, Vinayaka Nagar, Nagasandra Bangalore-560073, Karnataka, India
2- String Bio Private Limited, Centre for Cellular and Molecular Platforms, Bangalore-560065, Karnataka, India
3- VIT School of Agricultural Innovations and Advanced Learning (VAIAL), Vellore Institute of Technology, Vellore- 632014, Tamil Nadu, India
4- School of Biosciences and Technology (SBST), Vellore Institute of Technology, Vellore-632014, Tamil Nadu, India

*Dr. Ezhilkani Subbian- Corresponding Author

Dr. Theivasigamani Parthasarathi- Co-corresponding Author:

Email: subbiane@stringbio.com; parthasarathi.t@vit.ac.in

Supporting Methods

Multilocation Trials

To understand the efficacy of microbial biostimulant on improving grain yield under different agro-ecological regions, multilocation trial was conducted at Jhansi, Uttar Pradesh, India (GPS coordinate: 25.5101° N, 78.5411° E), Jabalpur, Madhya Pradesh, India (GPS coordinate: 23.2152° N, 79.9601° E) and Mandya, Karnataka, India (GPS coordinate: 12.5690° N, 76.8107° E). Crop management practices recommended for each location was followed. Seed variety used were Pusa Basmati 1121 (Jhansi, Uttar Pradesh, India), JR-206 (Jabalpur, Madhya Pradesh, India) and MTU-1001 (Mandya, Karnataka, India). Microbial biostimulant applications at the rate of 10mL/L were performed at three times during the crop growth (seedling root dipping during transplantation, foliar application during tillering stage and panicle development stage). Control plots received water sprays.

Isolation and confirmation by microbial cells in rice roots and leaves by quantitative polymerase chain reaction

The presence of *M. capsulatus* in rice roots were evaluated by seedling root dip treatment and foliar application followed by re-isolation. Roots of paddy seedlings (25 days old) were dipped in microbial biostimulant suspension for 20 min. For control plants, the roots were dipped into water. Treated seedlings were transplanted in pot containing soil + farmyard mannure (1:1). Another set of plants were sprayed at 10ml/L dose of microbial biostimulant whereas control plants received water spray. Plants were maintained at the greenhouse facility. One week after transplantation, the seedlings were carefully uprooted, and roots were gently washed with running tap water to remove all soil particles. Root and leaves tissues were selected for isolation and root/leaves samples were surface sterilized by 70% ethanol for 2 min, and then samples were washed thrice with sterile water. Surface sterilized roots/leaves were cut into small pieces or segments of 0.5 to 1cm in size aseptically using a sterile blade. Root segments of 15 to 20 numbers were suspended in conical flask containing 20ml of nitrate mineral salts (NMS) media. The flasks were sealed with suba-seal and head spaces were filled with methane (0.2 bar), incubated @ 45˚ C at 180rpm in an orbital shaker. At 4th day of incubation, 100µL of cultures were transferred to 20 ml of fresh NMS media with methane as carbon source and continued incubation to enrich the microbial culture. The enriched cultures were used to RT-qPCR to reconfirm the culture identity.

Genomic DNA isolated from the cultures of control and microbial biostimulant treated paddy samples along with positive control (pure genomic DNA isolated from bacterial strain) were

subjected for strain specific RT-qPCR using *MopB*- primers followed by High resolution melt curve analysis developed in house. All PCR assays were performed as single-tube assays in triplicates in 0.1-mL strip tubes and caps using Quant studio 3^{TM} system (Applied Biosystems Inc, USA). Each 10 µL reaction mixture contained 0.5 µL of both forward and reverse primers (10pM), DNA template (100ng), 5µL of Melt doctor reagent^{TM} (Applied Biosystems; Catalog number:4425557) and 3.5 µL of MilliQ water. Each sample was incubated for five minutes at 95 °C, then was denatured for 10 seconds at 94 °C, annealed for 10 seconds at 60 °C, extended for 10 seconds at 72 °C for 40 cycles. A post-PCR HRM analysis was performed from 72 °C to 95 °C, increasing at 0.2 °C/step. The results were analyzed using high resolution melt software (Applied Biosystems Inc, USA).

Extraction and quantification of IAA

The IAA production by the *M. capsulatus* was quantified by growing cells in methane derived microbial biostimulant for 3 days at 45 °C in Nitrate Mineral Salt (NMS) broth supplemented with and without 5mM tryptophan. After incubation, the broth was centrifuged at 5000×g for 10 min, and IAA was quantified in the supernatant by high performance liquid chromatography (HPLC). The instrument (Model: 1260 Infinity II with quaternary pump, autosampler & VWD detector, Agilent Technologies) equipped with C_{18} symmetric reverse phase column (Poreshell 120- 4.6 × 250 mm, 4 µm, Agilent Technologies) was used for analysis. Separation was carried out in isocratic mode with mobile phase consisting of 0.1% acetic acid (A) and Acetonitrile (B) at a ratio of 40:60 A:B. The column temperature was maintained at 30°C with a flow rate of 0.5 ml/min. The total run time was 15 minutes. Data was extracted at 287 nm and peak area obtained from standards and samples were used to quantify the levels of IAA.

Soil and Plant Nutrient Analysis

The plant, grain and soil analysis methods were carried out as per Motsara and Roy (2008). A random sampling consisting of fiver plants per plot was taken for plant and grain nutrient analysis. Plant/grain samples (minimum from five tagged plants) were collected from each treatment at 90 DAT and they were cleaned and shade dried. Later the shade-dried samples were oven-dried at 60 ± 5º C for 24-48 hours. The samples were finely powdered using mixer grinder. The finely ground plant samples were used for analysis. Total nitrogen was estimated using micro Kjeldahl digestion and distillation method (Jackson, 1973). Digestion of plant samples were carried out with di-acid mixture. Exactly 0.5 g of powdered plant sample was pre-digested with 5 ml of concentrated HNO_3 and digested with a di-acid mixture (HNO3:HClO4 in the proportion of 9:4 ratio). Digested samples were diluted with distilled water and volume was made up to 100 ml. The same samples were preserved for P and K analysis.

To determine total phosphorus, vanadomolybdophosphoric yellow colour method was used and the phosphorus content in the digest was estimated using spectrophotometer (Tandon, 2005). Total potassium content in the digested samples was estimated using flame photometer (Tandon, 2005). To analyse micronutrients, 0.5g of 100 mm mesh powdered sample was digested with diacid (HNO3:HClO4 in 9:4) mixture in a digestion chamber. After complete digestion, it was filtered (Whatman No. 42) to a 25 ml volumetric flask and volume was made by thoroughly washing with deionized water. This sample was preserved for micronutrient estimation by using atomic absorption spectrophotometer.

The uptake of nutrients was estimated using below formula:

$$\text{Uptake (kg ha}^{-1}\text{)} = \frac{\text{Nutrient concentration (\%)} \times \text{Weight of dry matter (kg ha}^{-1}\text{)}}{100}$$

Soil analysis before and after trial was carried out to get the levels of initial and final macronutrient availability. For soil macronutrient analysis, soil samples from all the treatments were taken at 90 DAT. Samples were obtained from the surface (0 to 15 cm). The samples collected were shade dried, finely powdered and sieved using 2 mm sieve. Sieved soil sample was used for analysis. Available nitrogen was determined by modified alkaline potassium permanganate method (Sahrawat and Burford, 1982). To check the levels of available phosphorus, sample was extracted using 0.5 M sodium bicarbonate at pH 8.5 (Olsen et al., 1954). The intensity of colour developed by stannous chloride was measured in spectrophotometer at 660 nm. To determine the levels of available K, sample was extracted with neutral 1N ammonium acetate extract (Hanway and Hiedal, 1952) and the content was determined by flame photometer. The amount of nutrients is expressed as relative percentage to the controls.

References:

Hanway, J. J., and Heidal, H. (1952). Soil analysis methods as used in Iowa State College Soil Testing Laboratory. Iowa State College of Agriculture Bulletin. 57, 1-31.

Jackson, M. L. (1973). Soil chemical analysis prentice hall of India private limited. New Delhi, 498.

Motsara, M. R., and Roy, R. N. (2008). Food and Agriculture Organization of the United Nations. Guide to laboratory establishment for plant nutrient analysis. 204.

Olsen, S. R., Cole, C. V., and Watanabe, F. S. (1954). Estimation of available phosphorus in soils by extraction with sodium bicarbonate. USDA Circular No. 939, Washington DC: US Government Printing Office.

Sahrawat, K. L., and Burford, J. R. (1982). Modification of the alkaline permanganate method for assessing the availability of soil nitrogen in upland soils. *Soil Sci.* 133, 53-57.

Tandon, V. R., and Gupta, R. K. (2005). An experimental evaluation of anticonvulsant activity of Vitex-negundo. *Indian J. Physiol. Pharmacol*. 49, 199.
