# Supplementary material for: Methane-derived microbial biostimulant reduces greenhouse gas emissions and improves rice yield
Source: Front Plant Sci. 2024 Sep 5;15:1432460. doi: 10.3389/fpls.2024.1432460 (PMC11410644; doi:10.3389/fpls.2024.1432460)
Supplement: Supplementary file 2 [file DataSheet2.pdf]

# Methane-derived microbial biostimulant reduces greenhouse gas emission and improves rice yield

• Sarma Rajeev Kumar[1, 2] †, Einstein Mariya David[3,4]†, Gangigere Jagadish Pavithra[1], Gopalakrishnan Sajith Kumar [3,4], Kuppan Lesharadevi[3,4], Selvaraj Akshaya[1,2],Chavadi Bassavaraddi[1], Gopal Navyashree[1], Panakanahalli Shivaramu Arpitha[1], Padmanabhan Sreedevi[1,2], Khan Zainuddin[1], Saiyyeda Firdous [3], Bondalakunta Ravindra Babu[1], Muralidhar Udagatti Prashanth[1], Ganesan Ravikumar[1], Palabhanvi Basavaraj[1], Sandeep Kumar Chavana [1], Vinod Munisanjeeviah Lakshmi Devi Kumar[1,2], Theivasigamani Parthasarathi[3] and Ezhilkani Subbian[1,2]*

†- These authors contributed equally to this work

1. String Bio Private Limited, Vinayaka Nagar, Nagasandra Bangalore-560073, Karnataka, India
2. String Bio Private Limited, Centre for Cellular and Molecular Platforms, Bangalore- 560065, Karnataka, India
3. VIT School of Agricultural Innovations and Advanced Learning (VAIAL), Vellore Institute of Technology, Vellore- 632014, Tamil Nadu, India
4. School of Biosciences and Technology (SBST), Vellore Institute of Technology, Vellore-632014, Tamil Nadu, India

- *Dr. Ezhilkani Subbian- Corresponding Author
- Dr. Theivasigamani Parthasarathi- Co-corresponding Author:
- **Email:** subbiane@stringbio.com; parthasarathi.t@vit.ac.in

Fig. S1A

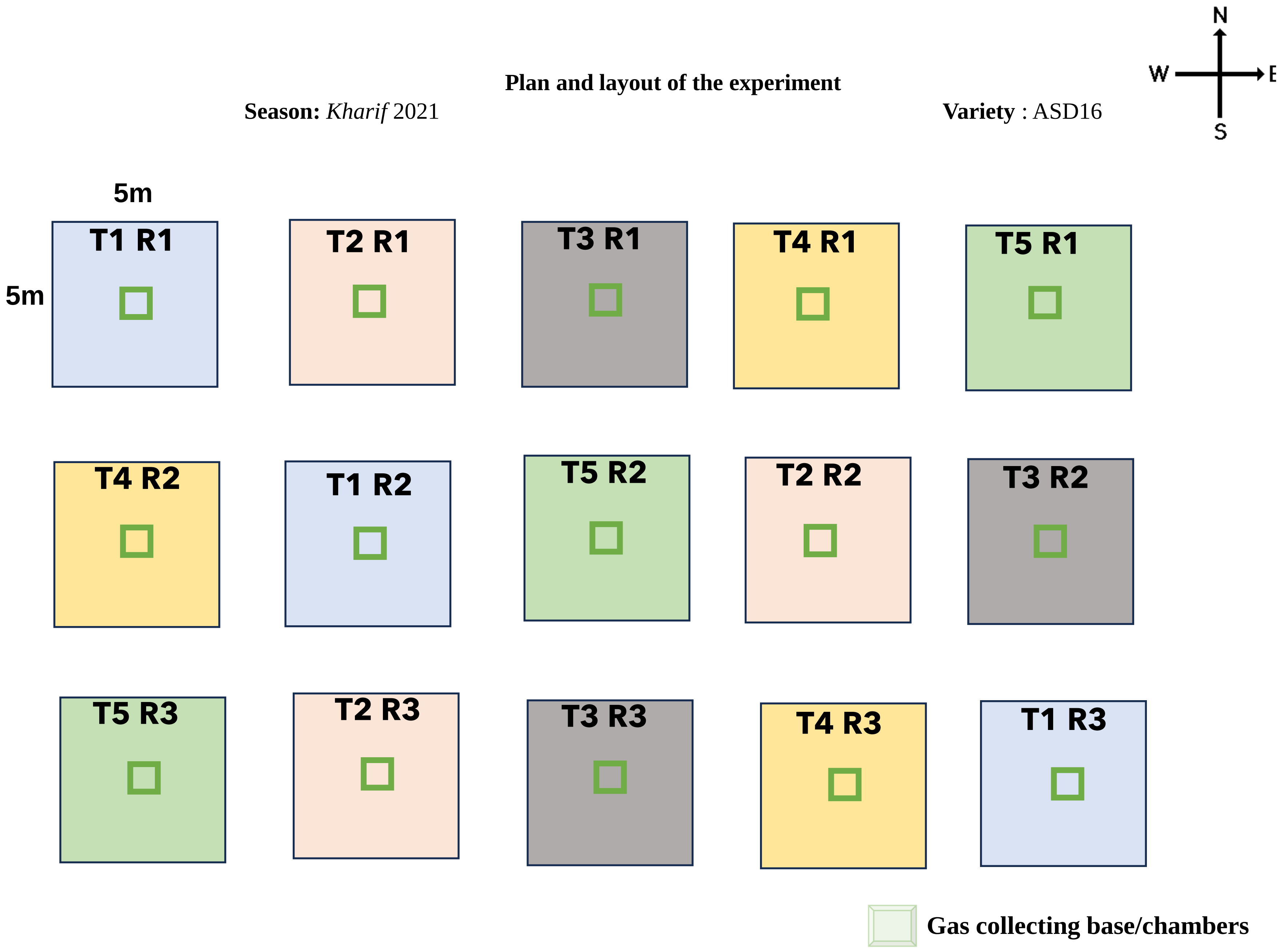


**Fig S1A-Experimental field layout for season I testing-** Experimental field layout for season I testing- Treatment details are below: Control (T1); 10 mlL/L dose of methane- derived microbial biostimulant under two different conditions (T2 & T3), T4 is 75% N control and T5 is 75%N+ 10 mL/L dose of methane- derived microbial biostimulant. First application was seedling root dipping for 20 min for T2, T3 and T5. For T3, second application was given as soil spray whereas in T3 & T5 it was foliar spray. T4 is 75% N control and T5 is 75%N+ 10 ml/L dose of methane derived microbial biostimulant. R1, R2 and R3 respectively corresponds to replication 1, 2 and 3. Small green box indicate position of gas collection base & chambers.

**Fig. S1B**

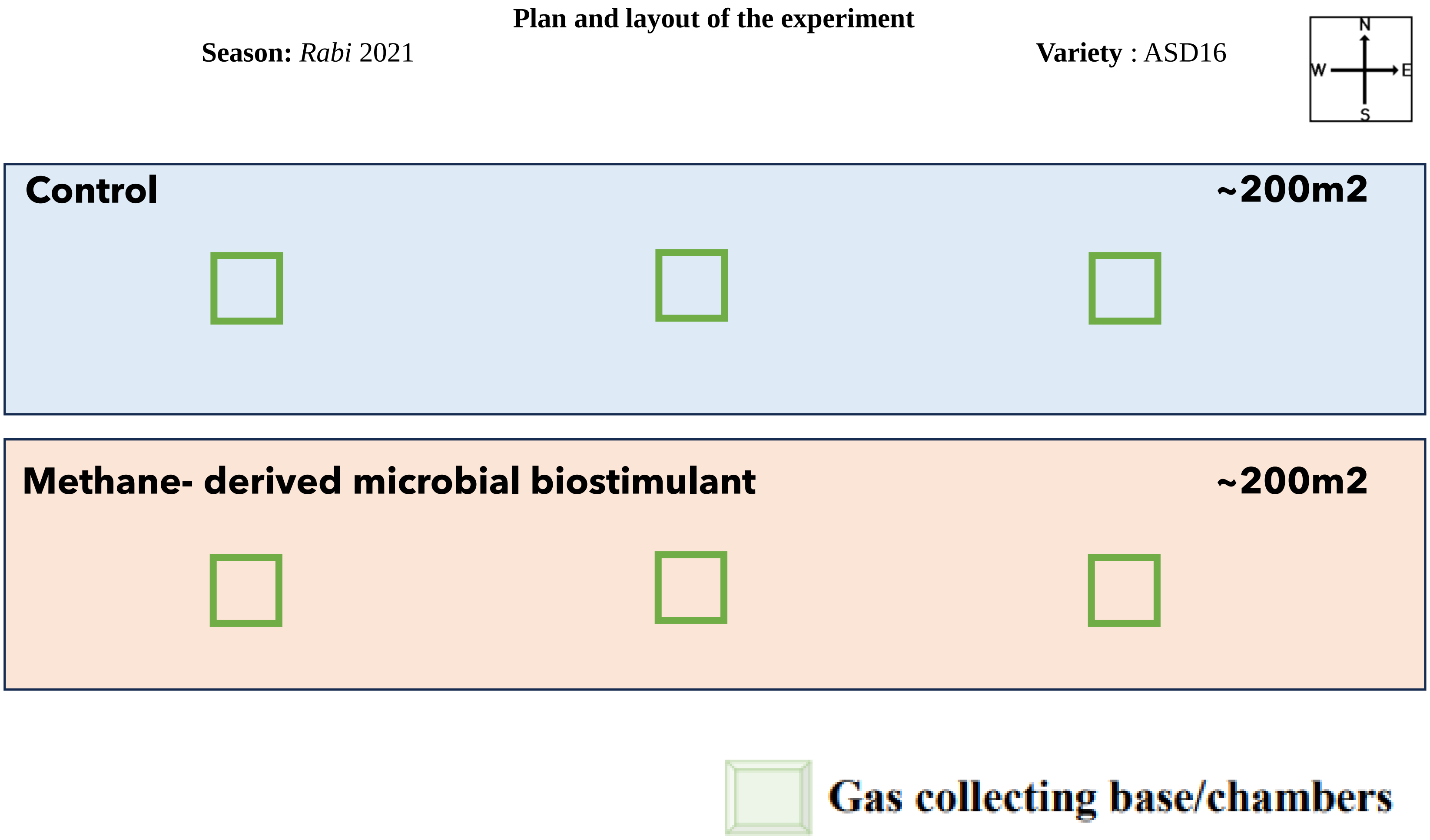


**Fig S1B- Experimental field layout for season II testing-** Treatment details are below: Control (T1) and methane- derived microbial biostimulant- 10mL/L (T2). Seedling root dipping during transplantation and foliar application during tillering stage and panicle development stage was followed for the application of microbial biostimulant. Control plants received water spray at the same time. Small square box indicates position of gas collection base & chambers.

**Fig. S1C**

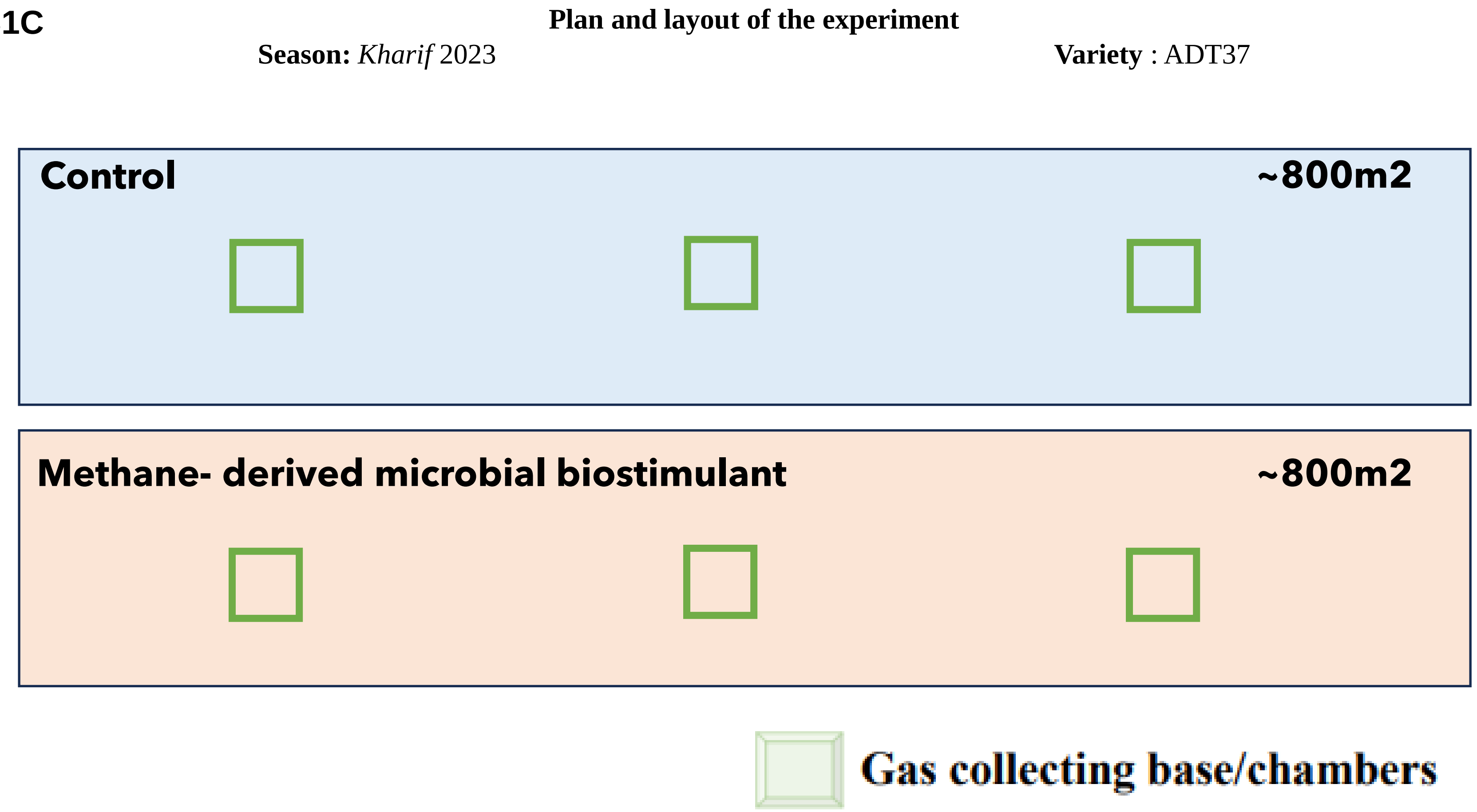


**Fig S1C- Experimental field layout for season III testing -** Treatment details are below: Control (T1) and methane- derived microbial biostimulant- 10mL/L (T2). Seedling root dipping during transplantation and foliar application during tillering stage and panicle development stage was followed for the application of microbial biostimulant. Control plants received water spray at the same time. Small square box indicates position of gas collection base & chambers.

S2A

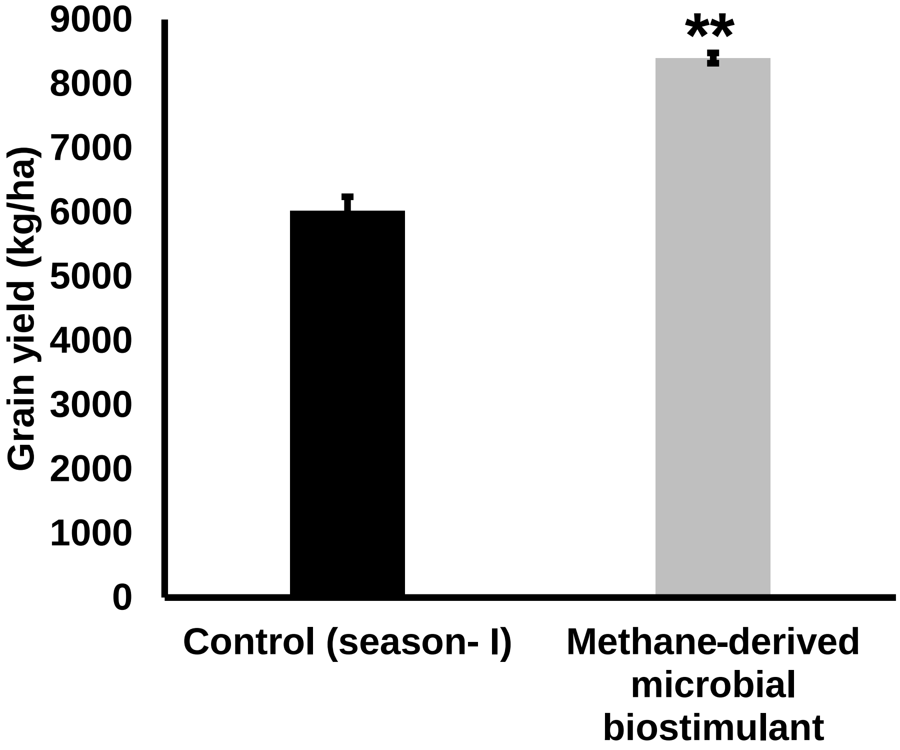


**Figure S2A- Influence of methane- derived microbial biostimulant on grain yield- S**econd application was given as soil spray instead of foliar spray. Differences were evaluated using the two-tailed Student's *t* test and significant differences at *P* < 0.01 is represented by "**".

S2B

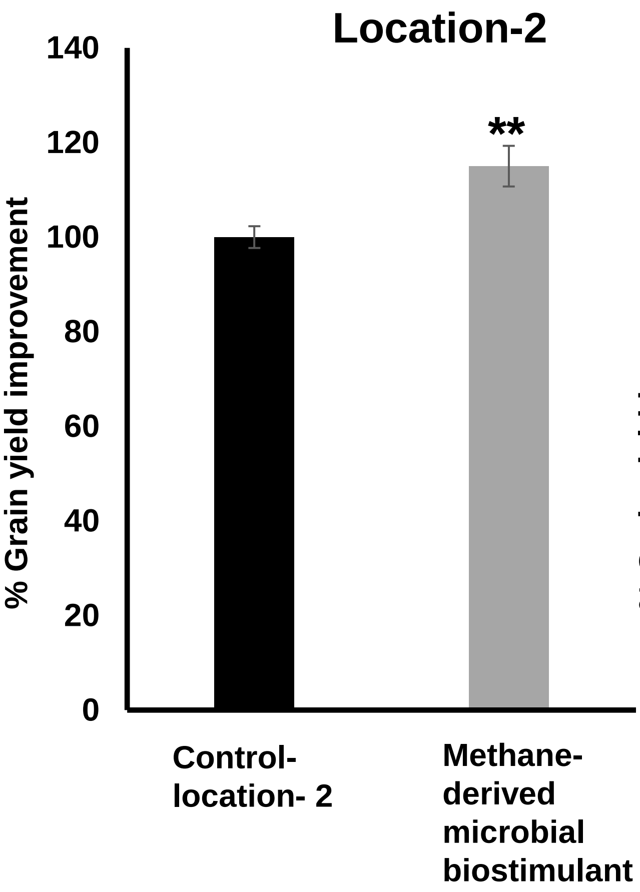


S2C

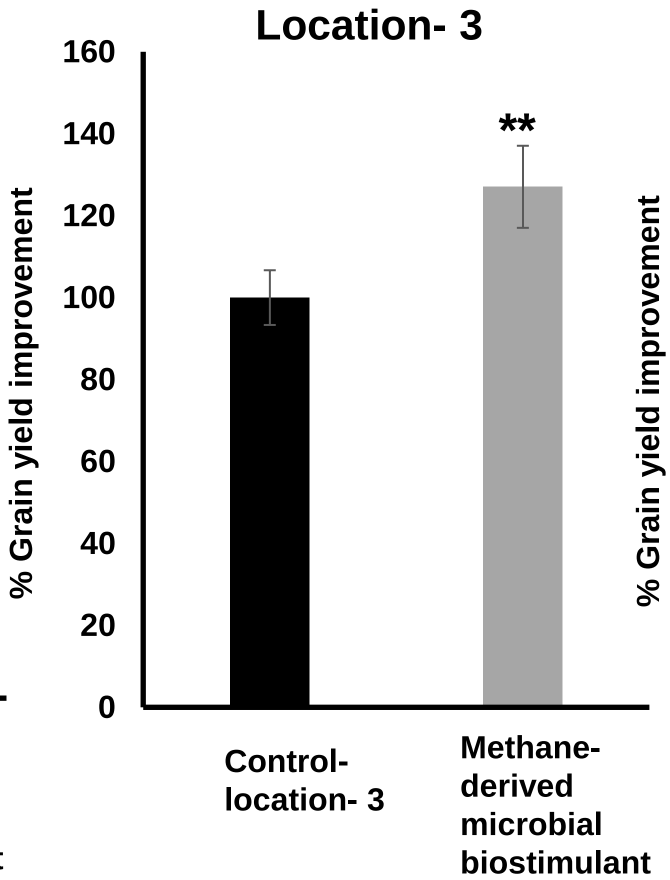


S2D

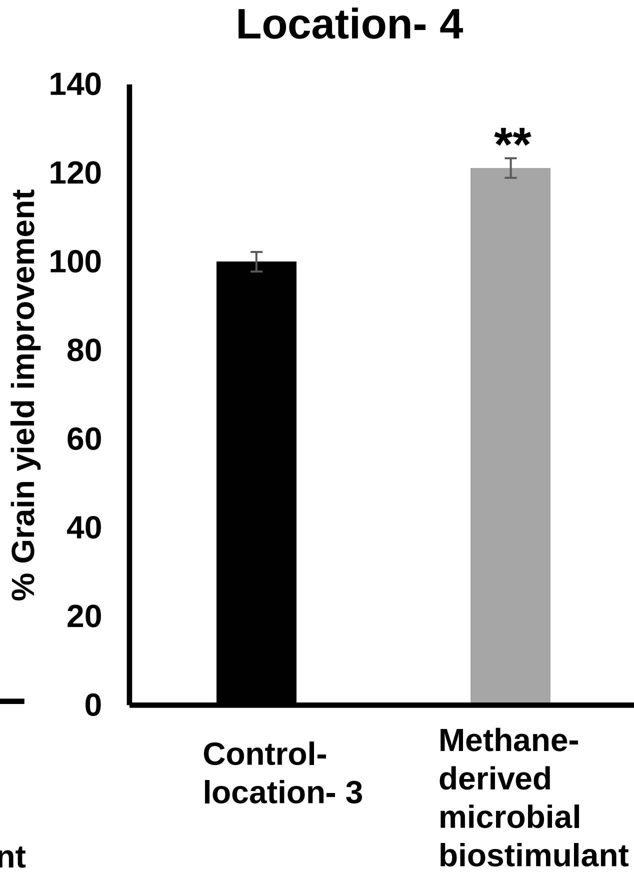


**Figure S2B-D- Multilocation microbial biostimulant validation data**- Grain yield improvement mediated by methane- derived microbial biostimulant under different agro-ecological locations in India. Differences were evaluated using the two-tailed Student's *t* test and significant differences *P* < 0.01 is represented by "**" .

S3A

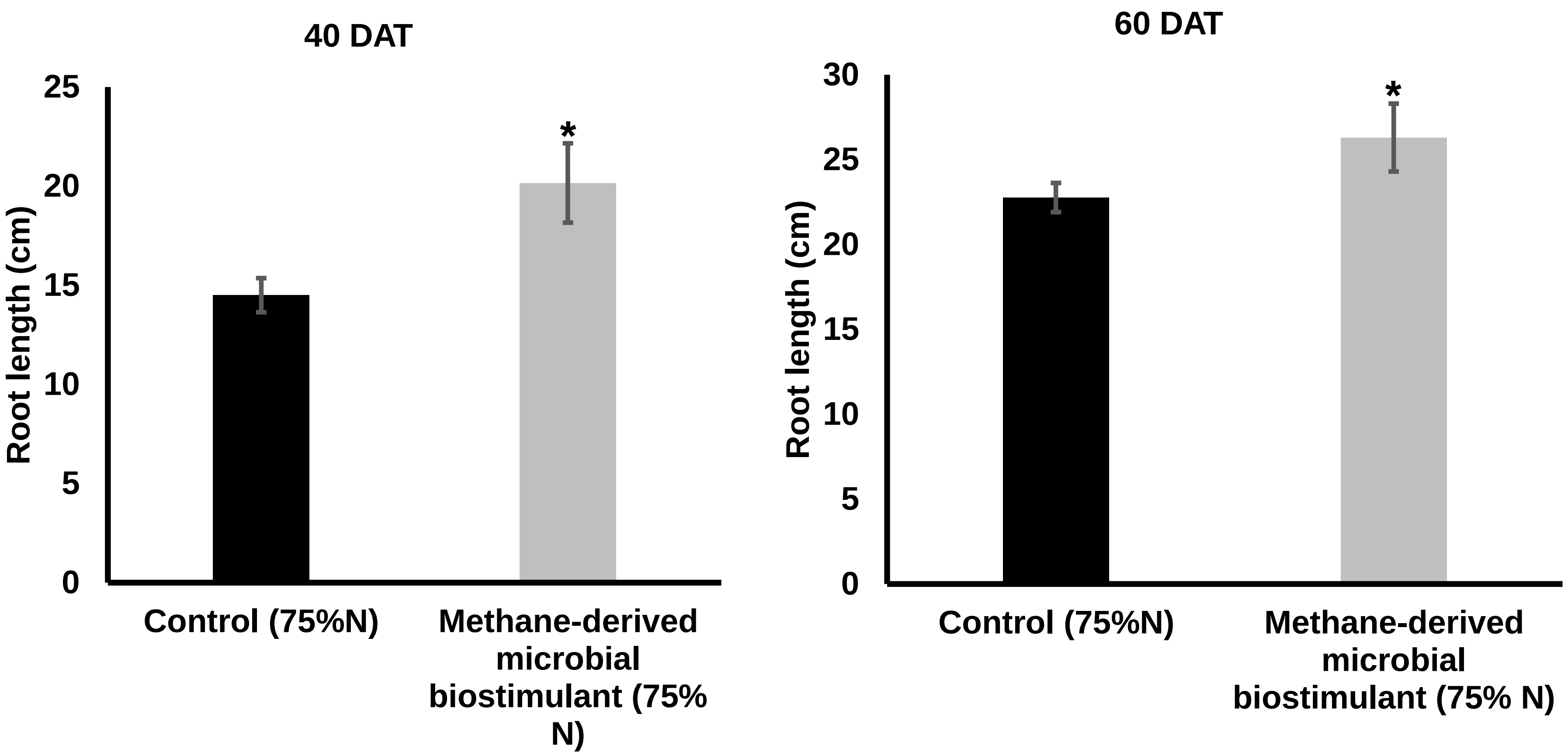


**Figure S3A- Effect of microbial biostimulant on root length at 75% N fertilizer-** Seedling root dipping was performed in paddy roots with microbial biostimulant. Five independent plants were uprooted from each treatment and root length was measured at 40 DAT and 60 DAT. Student's t-test Significant differences at *P* <0.05 is represented by "*".

S3B

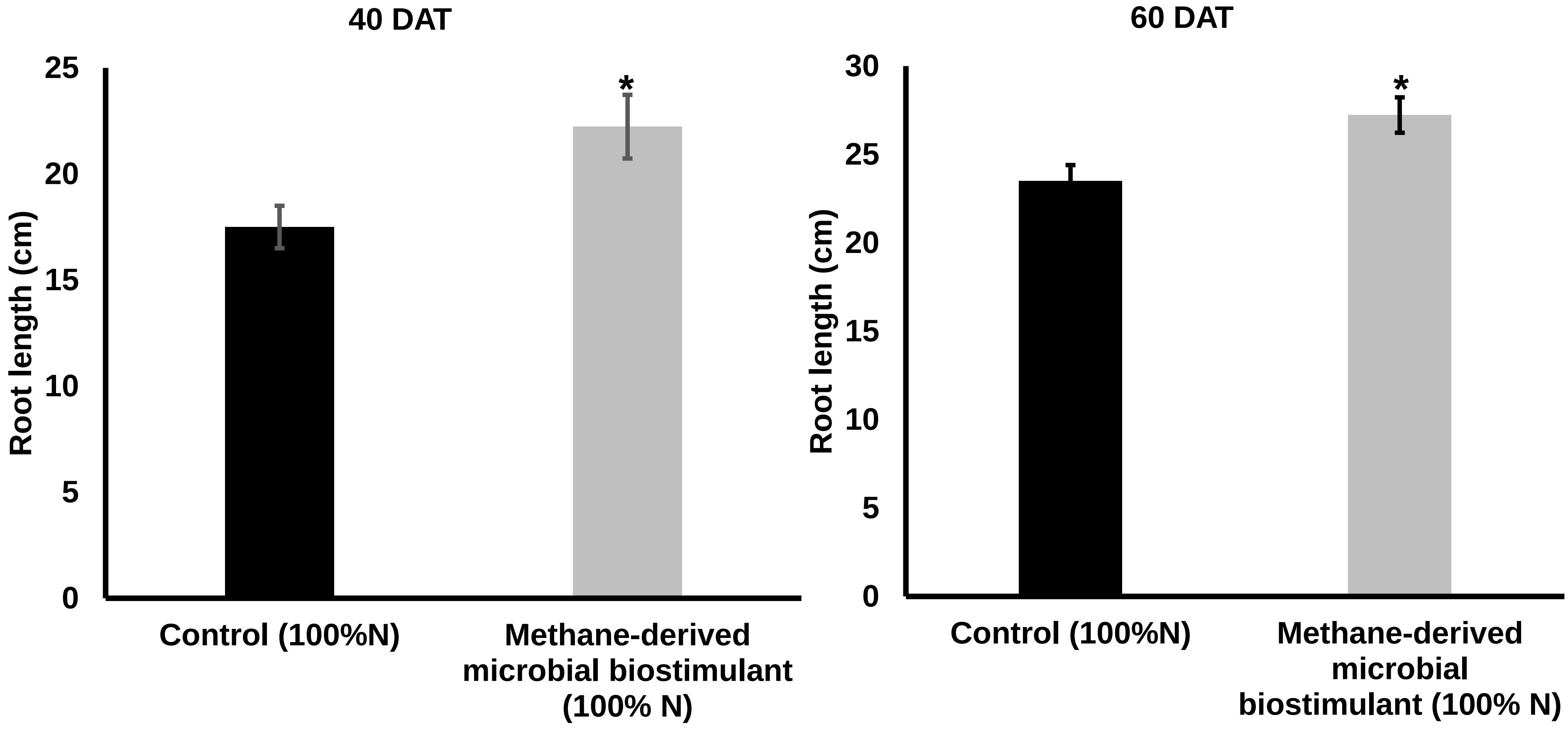


**Figure S3B- Effect of microbial biostimulant on root length at 100% N fertilizer-** Seedling root dipping was performed in paddy roots with microbial biostimulant. Five independent plants were uprooted from each treatment and root length was measured at 40 DAT and 60 DAT. Student's t-test Significant differences at *P* <0.05 is represented by "*".

S4A

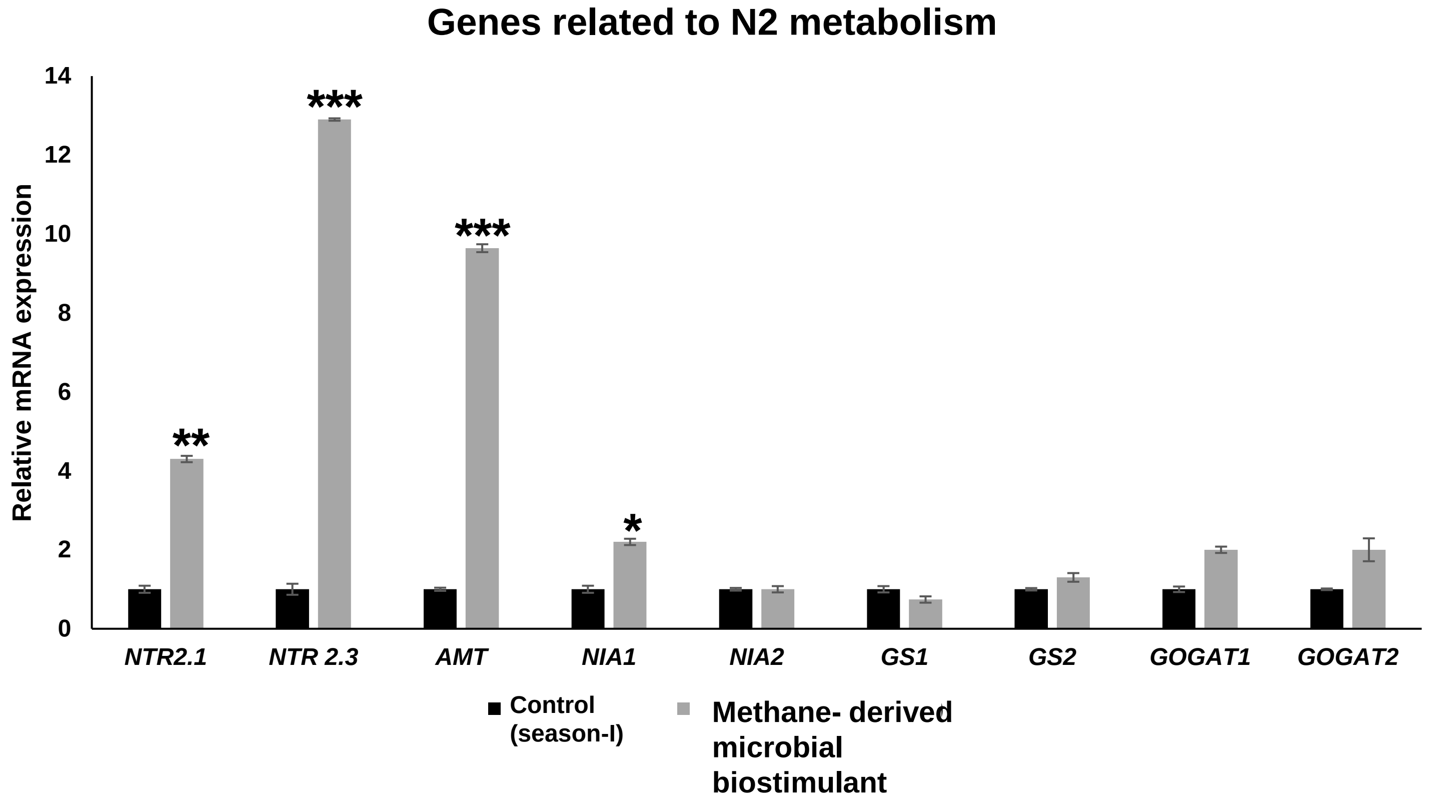


S4B

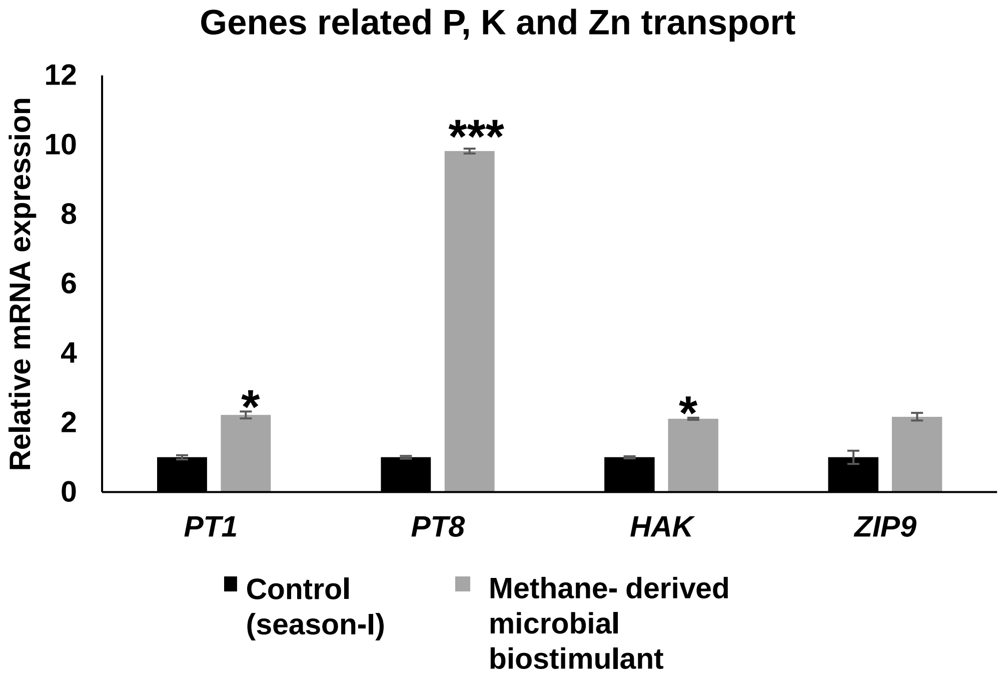


**Figure S4- Influence of methane- derived microbial biostimulant on expression of root nutrient uptake and transporter genes-** qRT-PCR analysis showing the expression of genes related to macronutrient transport and metabolism in roots of microbial biostimulant treated plants. Expression levels of genes were normalized to the endogenous reference gene actin and are represented relative to respective control roots, which was set to 1. Pooled root samples from control and microbial biostimulant treated roots used for RNA extraction. The results shown are from three independent experiments. Error bars indicate mean ± SE. Student's t-test: significant differences at $P < 0.05$, $P < 0.01$ and $P < 0.001$ are represented by *, ** and ***, respectively.

Nitrate transporter (*NRT*); Ammonium transporter (*AMT*); nitrate reductase (*NIA*); Glutamine synthetase (*GS*); glutamate synthase (*GOGAT*); Phosphate transporter (*PT*); High affinity potassium transporter (*HAK*); Zinc transporter (*ZIP*).

S5

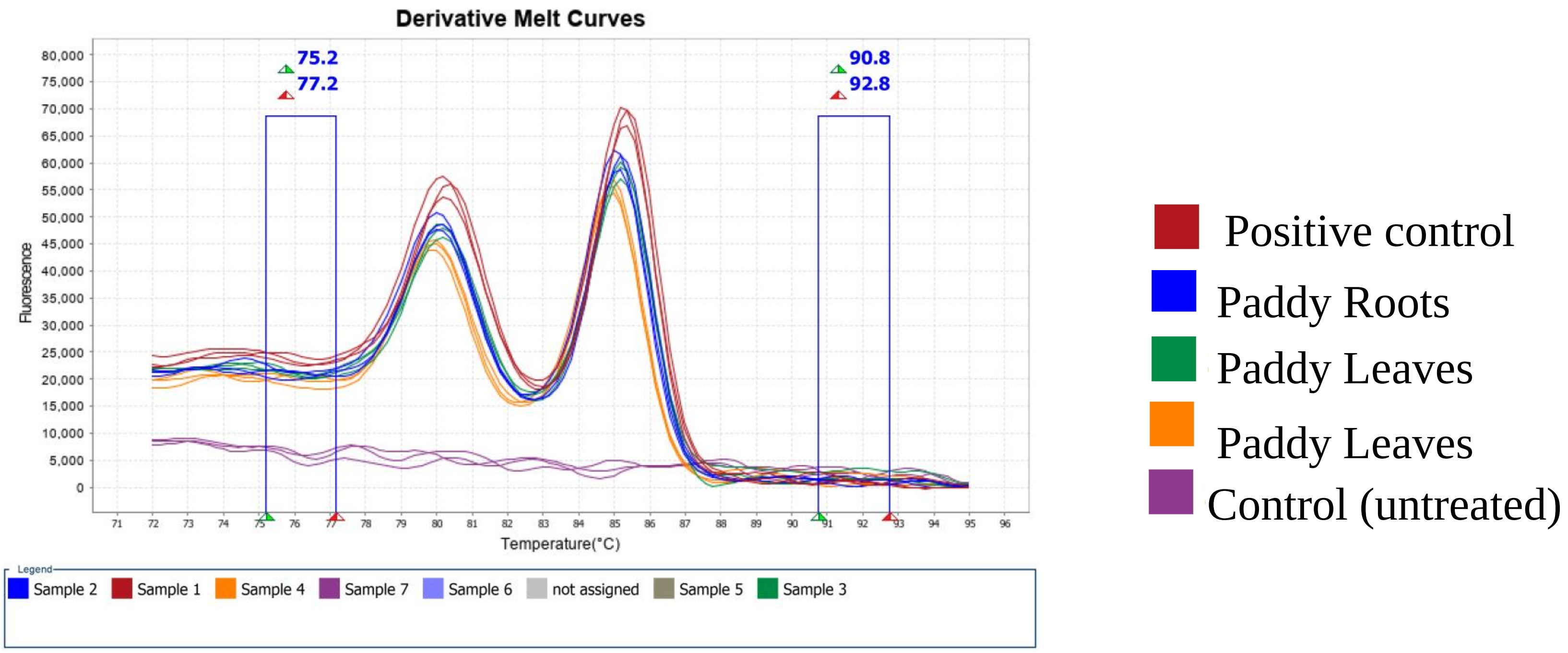


**Figure S5- qRT-PCR based derivative melt curve analysis showing the presence of *M. capsulatus* in paddy roots and leaves.**

Fig 6A

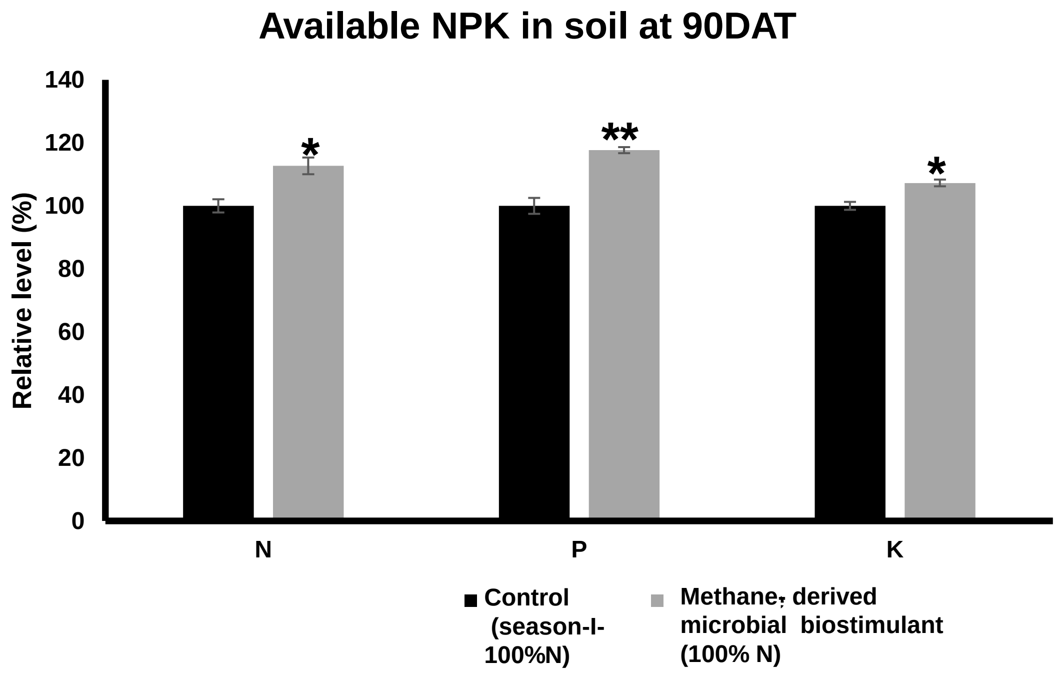


Fig 6B

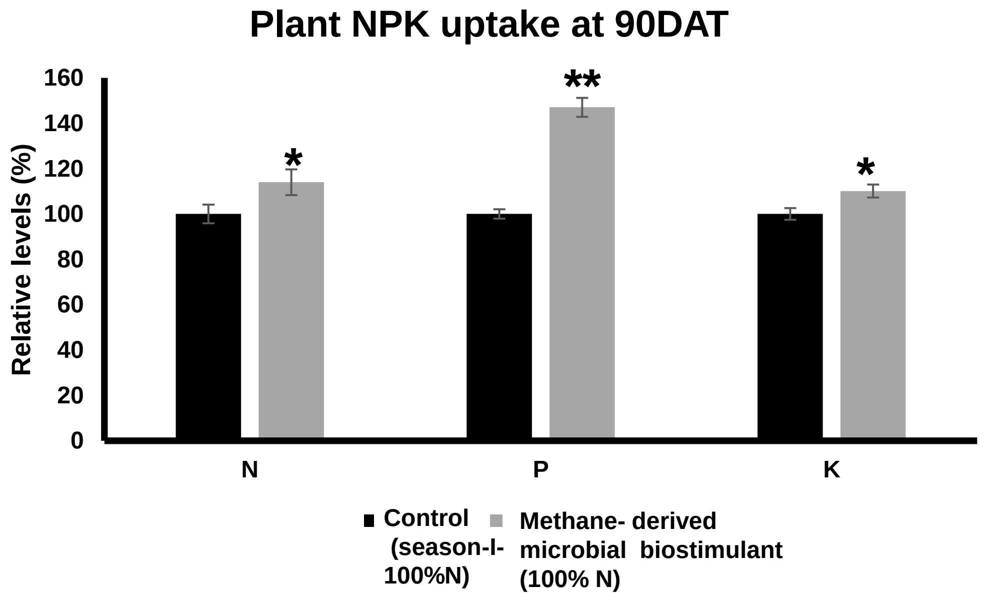


**Figure S6A&B- Soil and plant nutrient analysis-** Influence of microbial biostimulant on soil NPK levels (A) and plant NPK levels (B). Student's t-test: significant differences at $P < 0.05$ and $P < 0.01$ are represented by "*" and "**" respectively.

| Gene | Gene Accession No. | Forward Primer sequence (5' - 3') | Reverse Primer sequence (5' - 3') |
|---|---|---|---|
| *OsActin* | AB047313.1 | ACCATTGGTGCTGAGCGTTT | CGCAGCTTCCATTCCTATGAA |
| *OsPsaH* | Os05g0560000 | GAGGACATCGGCAACACCA | GCCCTTCTTGATCGGAAGCA |
| *OsFD1* | LOC_Os08g01380 | AGCAACAAGCTGGGAGACAG | GAGTAAGGCAGGTCGATCCC |
| *OsET* | LOC_Os04g33630 | GTGGGTGGCGGTGGCAAG | CACGACGGGCCTCAGCTC |
| *OsPsbD* | LOC_Osp1g00170 | CTGCTACTGCTGTTTTCT | GATGTTATGCTCTGCCTG |
| *OsPsbP* | Os07g0141400 | CACGGAGTTCATCGCCTACA | AAGCAAGAAGTCGACCTGGG |
| *OsPsbR3* | Os08g0200300 | GAGGTGGCATGTCAGTCGAT | TTTGCGCCGTACTTGTCAAC |
| *OsPsbS1* | LOC_Os01g64960 | GCTGTTCGGCTTCACCAAGG | ACGCCGGTCTCGATGTTGA |
| *OsCAB1R* | LOC_Os09g17740 | GTTCTCCATGTTCGGCTTCT | GACGAAGTTGGTGGCGTAG |
| *OsCAB2R* | LOC_Os01g41710 | TGTTCTCCATGTTCGGCTTCT | GCTACGGTCCCCACTTCACT |
| *OsCP24* | XM_015781325.2 | CCTTAACCTTAACGGCCATTTCC | CCCGCAGTAAAATCCTGAGC |
| *OsOEP3* | Os07g0544800 | AGCCGCTCATCGAGAAGAAG | GTACTTCTCCGCCTCTTCGG |
| *OsTLP* | LOC_Os08g39430 | GGAGGGAGACGGGGTGGG | TCAGAAAGGGAGGAGAGCCGT |
| *OsLHC2.1* | LOC_Os02g52650 | GGCGCGGTCGAACGAGCT | GTTTAGCCATTAGTCAAAGCAATC |
| *OsChlI* | LOC_Os03g36540 | CTTCACCGTCTGCAATGTAG | GATCTTAGGGTCGATGACGTT |
| *OsChlH* | LOC_Os03g20700 | GCACGGGAACTTGGCGTTTCATTA | ACATGTCCTGGAGCTGCTTCTCAT |
| *OsChlD* | LOC_Os03g59640 | TAGCACAGCTGTCAGAGTGGGTTT | TTGCCAGCCACCTCAAGTATCTCA |
| *OsHEMA* | AB011416.1 | GATGCAATCACTGCTGGAAAGCGT | CCATCTTGCCAGCACCAATCAACA |
| *OsHEMC* | LOC_Os02g07230 | GAGCTGTCTCATTCAGATCTGT | AGGGATTACAATAACCGTGGAA |
| *OsYGL13* | LOC_Os08g06630 | CTCTCCCACAAGATCAAGGATG | GTTTGTATGAGGGCTCATTTCC |
| *OsYGL8* | Loc_Os01g73450, | TGGATCTAACATGACACGCACCCA | ACTGTAACGGCATTCTTCTCCGGT |
| *OsRCA* | U74321.1 | CTCTTCGTGCCCGTGTTTAC | TCGGAGTTAGCGTCACCAAG |
| *OsRbcS2* | L22155.1 | AGTCTGGTGGCAACTAAGCC | GCACGGCCGGTAAAATCAAA |
| *OsRbcS3* | AK068555.1 | ACCATCTCAATGGCCTCTGC | TGTGTGCATATAGCCGGAGC |
| *OsRbcS4* | AK070257.1 | AACGTTAGGCAGGTGCAGTT | TGCAGCTTAACACGGACACA |
| *OsRbcS5* | AK099574.1 | GGAGTCCGGCGGAAACTAAG | GGAAACCAATGCAAGGTGGC |
| *OsHYR* | Os03g02650 | CCGAGGGCTTGATGATGAG | CGTAAGCCCATTTCAGGAATG |
| *OsMOC1* | AY242058 | CTGCTCCGGCTGCACTAC | CCACGCTGAGACGGAGAG |
| *OsSLR1* | Os03g0707600 | GAGTCGCTGCACTACTACTCC | GCGCGTGTGCCAGCCCAGCGT |
| *OsLAX2* | AB669025 | TTCTTGCCTCAGATTCCAAG | CCTCTGCATGTTATCTCCAC |
| *OsMADS57* | LOC_Os02g49840 | CAGATTATGTTGTCGGATGCTC | AAAGCAATAGAGAGTAAGCAGGGT |
| *OsHSF2AD* | LOC_Os03g06630 | CAGCAGGCACTTGGCACC | TTCTTGTCACGCTTTAGCCTGT |
| *OsCKX11* | Os08g0460600 | CAACGCAATCATTGACGCC | TTGCACCCCTCCCAAATGT |
| *OsRGN1* | LOC_Os01g49160 | CGGCTACACCGACCAGGAG | CGCGATGATGGACCACCT |
| *OsNOG1* | MF687920 | TCCGACTTACAATGAACAC | GGTAGCAGGACTCCACTT |
| *OsSPL9* | LOC_Os05g33810 | AGATGGGCAGGTGATTAT | TGTGGGAGAGCTTTAGTC |
| *OsIPA1* | LOC_Os08g39890 | CGGTCGACTAGCTGCATCTGTTGG | CATCGTGTTGCTGGTTTGGTCGAAG |
| *OsSPL14* | Os08g0509600 | CAAGGGTTCCAAGCAGCGTAA | TGCACCTCATCAAGTGAGAC |
| *OsLC2* | AK101341 | AGCATCAGCTTTGGACGAGGA | CAGTTGGTGGAATAGAGCCAGAAT |
| *OsVIL2* | XM_015764905.2 | GGAGTATGCTTTCCGGATCA | GTGGGAAACAACATGTGCAG |
| *OsGRF4* | LC333011.1 | GAAAGCCTGTGGAAACGCA | CAACGCCGAGCCAAATGAG |
| *OsNRT2.1* | NM_001401744.1 | CTTCACGTCGTCGAGGTACT | CACTCGGAGCCGTAGTAGTG |
| *OsNRT2.3A* | XM_015773038.2 | CGCTGCTGCCGCTCATCCG | CCGTGCCCATGGCCAGAC |
| *OsNIA1* | XM_015767224.2 | TCAAGGTGTGGTACGTGGTG | CGAGGTCATAGCCCATCTTC |
| *OsNIA2* | NM_001422594.1 | TGTACCAGGTCATCCAGTCG | CGATGACGTACCACACCTTG |
| *OsAMT1* | AF001505.2 | GGTTTCTCTCCCTCTCCGAT | CCACCTTCACACCACACATT |
| *OsGS1* | AB180688.1 | TGTTTCTCCTCATCCCTGC | TCACAGTCCTCGCTTTGC |
| *OsGS2* | X14246.1 | GGAGAGGTCATGCCTGGTCAGT | ACTACACCAGCCTGCTCCGTTA |
| *OsGOGAT1* | XM_015793761.2 | GTGCAGCCTGTTGCAGCATAAA | CGGCATTTCACCATGCAAATC |
| *OsGOGAT2* | NM_001402625. | CCTGTCGAAGGATGATGAAGGTGAAA CC | TGCATGGCCCTACTATCTTCGCATCA |
| *OsPT1* | AF536961.1 | CGCTTCCGTACGAGTGGTAGT | GGTTCTTTCAAATCCAGGGAAA |
| *OsPT8* | AF536968.1 | AGAAGGCAAAAGAAATGTGTGTTAAAT | AAAATGTATTCGTGCCAAATTGCT |
| *OsHAK1* | NM_001402365.1 | GTTGATGATGCTGATGTTGGAAG | CCAACACTTTCAGCTGAAAC |
| *OsZIP9* | NM_001420533.1 | CATCAGTTCTTCGAAGGGATAGG | TGTGGTTAGCGAGAAGAAGATG |
| *McMopB* | AF031148.1 | ACAGGCCGAAGAGACTTTCA | GGTGGTGTTGCCTTCGTA AT |

**Supplementary Table 1- List of oligonucleotide primers used in this study.**

| Sample | IAA concentration (mg/L) |
|---|---|
| Microbial biostimulant without Tryptophan | 0 |
| Microbial biostimulant with Tryptophan | 1.83-3.61 |

**Table S2- Indole acetic acid levels observed in microbial biostimulant grown in presence or absence of Tryptophan.**

| Season | Treatment | $CH_4$ flux (g/ha/h) | | | | | | | | | |
|---|---|---|---|---|---|---|---|---|---|---|---|
| Season | Treatment | 10 DAT | 20 DAT | 30 DAT | 40 DAT | 50 DAT | 60 DAT | 70 DAT | 80 DAT | 90 DAT | |
| Season II | Control | 31.33±3.38 | 28.67 ±3.84 | 70 ± 1.53 | 109.66 ± 4.7 | 123.33 ± 6.33 | 64 ± 4.62 | 34.2 ± 0.9 | 22.43 ± 1.68 | 9.86 + 0.61 | |
| | Methane - derived microbial biostimulant | 23.66 ± 1.2 | 35.66 ± 1.42 | 53.66 + 1.76 | 71.33 ± 4.98 | 61.73 ± 1.92 | 49.46 ± 4.66 | 23.13 ± 1.73 | 15.73 ± 1. 07 | 5.80 ± 0.45 | |
| | | $N_2O$ flux (g/ha/h) | | | | | | | | | |
| | Control | 0.76 ± 0.02 | 0.83 ± 0.07 | 0.78 ± 0.05 | 2.67 ± 0.08 | 1.32 ± 0.05 | 1.82 ± 0.03 | 3.91 ± 0.25 | 3.91 ± 0.11 | 2.14 ± 0.15 | |
| | Methane-derived microbial biostimulant | 0.41 ± 0.07 | 0.55 ± 0.03 | 0.57 ± 0.03 | 0.82 ± 0.04 | 0.51 ±0.06 | 0.69 ± 0.04 | 2.07 ± 0.33 | 2.65 ± 0.13 | 1.47 + 0.08 | |
| | | | | | | | | | | | |
| | | $CH_4$ flux (g/ha/h) | | | | | | | | | |
| Season | Treatment | 10 DAT | 20 DAT | 30 DAT | 40 DAT | 50 DAT | 60 DAT | 70 DAT | 80 DAT | 90 DAT | 100 DAT |
| Season III | Control | 59.33 ± 1.86 | 44 ± 1.73 | 86.66 ± 0.88 | 103 ± 2.31 | 128 ± 2.65 | 149.33 ± 1.45 | 92.33 ± 3.28 | 49 ± 1.15 | 36.66 ± 1.45 | 28.33 ± 1.45 |
| | Methane-derived microbial biostimulant | 37.33 ± 1.20 | 26.33 ± 0.88 | 54.33 ± 1.45 | 47.66 ± 2.96 | 58 ± 3.46 | 72.66± 2.46 | 47± 1.53 | 16.33± 1.45 | 20.33± 0.88 | 12.66±0.67 |
| | | $N_2O$ flux (g/ha/h) | | | | | | | | | |
| | Control | 1.28±0.03 | 0.94 ± 0.02 | 0.76 ± 0.02 | 1.05 ± 0.03 | 1.13 ± 0.03 | 1.28 ± 0.07 | 1.79 ± 0.04 | 4.72 ± 0.03 | 2.70 ± 0.03 | 3.46 ± 0.26 |
| | Methane-derived microbial biostimulant | 0.58 ± 0.03 | 0.68 ± 0.03 | 0.45 ± 0.02 | 0.40 ± 0.02 | 0.62 ± 0.03 | 0.68 ± 0.02 | 0.66 ± 0.01 | 2.2 ± 0.2 | 1.06 ± 0.11 | 1.25 ± 0.03 |

**Table S3A- Temporal $CH_4$ and $N_2O$ emission from control and microbial biostimulant- treated paddy fields collected during season II and season III trials**

| Season | Treatment | Average $CH_4$ emission (kg/ha/season) | Average $N_2O$ emission (kg/ha/season) |
|---|---|---|---|
| Season I Kharif 2022 | Control (Season-I) | 213.65 | 16.24 |
| | Methane- derived microbial biostimulant | 82.43 | 10.73 |
| Season II Rabi 2022 | Control (Season-II) | 131.60 | 5.32 |
| | Methane- derived microbial biostimulant | 90.72 | 2.80 |
| Season III Kharif 2023 | Control (Season-III) | 226.97 | 3.10 |
| | Methane- derived microbial biostimulant | 117.83 | 1.54 |

**Table S3B- Average $CH_4$ and $N_2O$ emission from control and microbial biostimulant- treated paddy fields.**

| | | | | $CH_4$ emission | | | | $N_2O$ emission | | | |
|---|---|---|---|---|---|---|---|---|---|---|---|
| Season | Treatment | Grain yield (kg/ha) | % improvement in grain yield over control | Average $CH_4$ emission (kg/ha/season) | % reduction in $CH_4$ emission over control | $CO_2$ eq $CH_4$ emission at 25- year time period (kg/ha/season) | Yield-scaled $CO_2$-eq emission (kg $CO_2$-eq/t) | Average $N_2O$ emission (kg/ha/season) | % reduction in $N_2O$ emission over control | $CO_2$ eq $N_2O$ emission at 25- year time period (kg/ha/season) | Yield-scaled $CO_2$-eq emission (kg $CO_2$-eq/t) |
| Season I Kharif 2022 | Control (Season-I) | 6024 | - | 213.65 | - | 17946.43 | 2979.16 | 16.24 | - | 4839.52 | 803.37 |
| | Methane- derived microbial biostimulant | 8004 | 32.87 | 82.43 | -61.42 | 6924.29 | 865.10 | 10.73 | -33.93 | 3197.54 | 399.49 |
| Season II Rabi 2022 | Control (Season-II) | 5015 | - | 131.60 | - | 11054.40 | 2204.27 | 5.32 | - | 1585.36 | 316.12 |
| | Methan-e derived microbial biostimulant | 6997 | 39.52 | 90.72 | -31.06 | 7620.48 | 1089.11 | 2.80 | -47.37 | 834.40 | 119.25 |
| Season III Kharif 2023 | Control (Season-III) | 5081 | - | 226.97 | - | 19065.60 | 3752.33 | 3.10 | - | 923.80 | 181.81 |
| | Methane- derived microbial biostimulant | 7058 | 38.91 | 117.83 | -48.09 | 9897.41 | 1402.30 | 1.54 | -50.32 | 458.92 | 65.02 |

**Table S4- Effect of methane-derived microbial biostimulant on yield-scaled CO2-eq emission in rice-** Yield-scaled CO2e-emission of $CH_4$ and $N_2O$ were found to be significantly lower in methane-derived microbial biostimulant treatment compared to controls.

Conversion factor for $CH_4$ (1kg of $CH_4$= 84Kg of $CO_2$ equivalent) and $N_2O$ (1Kg of $N_2O$= 298Kg of $CO_2$ equivalent) were taken from climatechangeconnection.org/emissions/co2-equivalents/

| | | | |
|---|---|---|---|
| Methane emission (million metric ton) | 380 | | |
| Methane emission from paddy - 10% contribution (million metric ton) | 30.4 | | |
| Global land under paddy cultivation (million ha) | 162 | | |
| Reduction in methane emission from methane-derived microbial biostimulant (percentage per ha) | 60% | | |
| Global targeted methane reduction per annum (2% of total - COP26 target) million metric tonnes | 7.60 | | |
| Percentage of global paddy cultivation area targeted formethane-derived microbial biostimulant application | 10.00% | 30.00% | 50.00% |
| Actual paddy cultivated area targeted for methane-derived microbial biostimulant application (million ha) | 16.2 | 48.6 | 81 |
| Total methane emission from target area (million metric tonnes) | 3.04 | 9.12 | 15.2 |
| Methane emission from methane-derived microbial biostimulant use in target area (million metric tonnes) | 1.824 | 5.472 | 9.12 |
| Percentage of global methane target achieved | 24% | 72% | 120% |

**Table S5- $CH_4$ emission reduction from paddy field using methane-derived microbial biostimulant to meet COP26 target for methane reduction by 2030.**
